# The Sixth Mass Extinction and Amphibian Species Sustainability Through Reproduction and Advanced Biotechnologies, Biobanking of Germplasm and Somatic Cells, and Conservation Breeding Programs (RBCs)

**DOI:** 10.3390/ani14233395

**Published:** 2024-11-25

**Authors:** Robert K. Browne, Qinghua Luo, Pei Wang, Nabil Mansour, Svetlana A. Kaurova, Edith N. Gakhova, Natalia V. Shishova, Victor K. Uteshev, Ludmila I. Kramarova, Govindappa Venu, Mikhail F. Bagaturov, Somaye Vaissi, Pouria Heshmatzad, Peter Janzen, Aleona Swegen, Julie Strand, Dale McGinnity

**Affiliations:** 1 Sustainability America, Sarteneja, Corozal District, Belize 91011, Belize; 2 Hunan Engineering Technology Research Center for Amphibian and Reptile Resource Protection and Product Processing, College of Biological and Chemical Engineering, Changsha University, Changsha 410022, China; dani2017@126.com; 3 Hunan Engineering Laboratory for Chinese Giant Salamander’s Resource Protection and Comprehensive Utilization, School of Biological Resources and Environmental Sciences, Jishou University, Jishou 416000, China; wangpei0229@126.com; 4 Fujairah Research Centre, University of Science and Technology of Fujairah, Fujairah P.O. Box 2202, United Arab Emirates; nabil.mansour@frc.ae; 5 Institute of Cell Biophysics, Russian Academy of Sciences, PSCBR RAS, Pushchino 142290, Moscow Region, Russia; sakaurova@mail.ru (S.A.K.); gakhova@gmail.com (E.N.G.); cryopreservation@list.ru (N.V.S.);; 6 Institute of Theoretical and Experimental Biophysics, Russian Academy of Sciences, Pushchino 142290, Moscow Region, Russia; luda_kramarova@rambler.ru; 7 Centre for Applied Genetics, Department of Zoology, Jnana Bharathi Campus, Bangalore University, Bengaluru 560056, Karnataka, India; venugcaecilian@gmail.com; 8 IUCN/SSC/Athens Institute for Education and Research/Zoological Institute RAS, St. Petersburg 199034, Northern Region, Russia; bbigmojo@mail.ru; 9 Leningrad Zoo, St. Petersburg 197198, Northern Region, Russia; 10 Department of Biology, Faculty of Science, Razi University, Baghabrisham, Kermanshah 57146, Iran; s.vaissi@razi.ac.ir (S.V.); pheshmatzad@gmail.com (P.H.); 11 Department of Fisheries, Faculty of Fisheries and Environmental Sciences, Gorgan University of Agricultural Sciences and Natural Resources, Gorgan 49138, Iran; 12 Justus-von-Liebig-Schule, 47166 Duisburg, Germany; pjanzen@gmx.de; 13 School of Environmental and Life Sciences, College of Engineering, Science and Environment, University of Newcastle, Callaghan, NSW 2308, Australia; aleona.swegen@newcastle.edu.au; 14 Department of Chemistry and Bioscience, Aalborg University, Fredrik Bajers Vej 7K, 9220 Aalborg Ost, Denmark and Randers Regnskov, Torvebryggen 11, 8900 Randers C, Denmark; js@biosfaeren.dk; 15 Ectotherm Department, Nashville Zoo at Grassmere, Nashville, TN 37211, USA; dmcginnity@nashvillezoo.org

**Keywords:** mass extinction, COP 16, COP 28, reproduction technologies, intergenerational justice, de-extinction, climate catastrophe, assisted evolution, terraforming, space colonization

## Abstract

Primary themes in intergenerational justice are a healthy environment, the perpetuation of Earth’s biodiversity, and the sustainable management of the biosphere. However, the current rate of species declines globally, ecosystem collapses driven by accelerating and catastrophic global heating, and a plethora of other threats preclude the ability of habitat protection alone to prevent a cascade of amphibian and other species mass extinctions. Reproduction and advanced biotechnologies, biobanking of germplasm and somatic cells, and conservation breeding programs (RBCs) offer a transformative change in biodiversity management. This change can economically and reliably perpetuate species irrespective of environmental targets and extend to satisfy humanity’s future needs as the biosphere expands into space. Currently applied RBCs include the hormonal stimulation of reproduction, the collection and refrigerated storage of sperm and oocytes, sperm cryopreservation, in vitro fertilization, and biobanking of germplasm and somatic cells. The benefits of advanced biotechnologies in development, such as assisted evolution and cloning for species adaptation or restoration, have yet to be fully realized. We broaden our discussion to include genetic management, political and cultural engagement, and future applications, including the extension of the biosphere through humanity’s interplanetary and interstellar colonization. The development and application of RBCs raise intriguing ethical, theological, and philosophical issues. We address these themes with amphibian models to introduce the Multidisciplinary Digital Publishing Institute Special Issue, The Sixth Mass Extinction and Species Sustainability through Reproduction Biotechnologies, Biobanking, and Conservation Breeding Programs.

## Simple Summary

Primary themes in intergenerational justice are a healthy environment, the perpetuation of Earth’s biodiversity, and the sustainable management of the biosphere. These goals demand transformative changes to biodiversity management, especially when considering the predicted sixth mass extinction. Reproduction and advanced biotechnologies, biobanks of germplasm and somatic cells, and conservation breeding programs (RBCs) provide a transformative change to perpetuate biodiversity irrespective of environmental targets, ecosystem collapses, and other sixth mass extinction drivers. Future potentials for RBCs include assisted evolution, species restoration, and the extension of the biosphere through interplanetary and interstellar colonization. We address these themes with amphibian models to introduce the MDPI Special Issue, The Sixth Mass Extinction and Species Sustainability through Reproduction and Advanced Biotechnologies, Biobanking, and Conservation Breeding Programs.

## 1. Introduction

The Earth’s biosphere is under threat from landscape modification, which, along with catastrophic global heating and other threats, is driving profound changes in ecosystems globally and accelerating the sixth mass extinction [1]. COP 15, 2022, ”Ecological Civilisation: Building a Shared Future for All Life on Earth”, and COP 16, 2024, “Sixteenth meeting of the Conference of the Parties to the Convention on Biological Diversity”, focus on direct habitat protection [2,3]. However, the COP 15 targets of protecting 30% of the world’s land and water and restoring 30% of degraded ecosystems by 2030 are unlikely to be met, with only 12 countries contributing only USD 413 million toward the 200 billion per year needed. Even if COP 15 targets were met, habitat modification, even in protected habitats, driven by catastrophic global heating, exotic predators and competitors, emergent pathogens, and major losses in prey and other taxa [3,4], will inevitably result in the mass extinction of many amphibian species in the wild [5,6,7,8]. Amphibians are the most threatened vertebrate group, where, as of the present date, the IUCN accesses 8011 species, with 36.4% (2912) having some degree of threat of extinction, with 16.8% (1263) species being Endangered and 10.0% (799) species Critically Endangered, and with these most threatened categories comprising ~23.5% of the 8772 described species on AmphibiaWeb. Only 52.3% of IUCN species are Near Threatened or of Least Concern, with 11.3% (908) being Data Deficient. Of further concern is that ~65% of populations are decreasing, with only ~40% being stable [9,10,11]. Similarly, rapid declines are also occurring across all terrestrial vertebrates [12].

COP 28, 2023 [13], emphasized the urgency of addressing catastrophic global heating as a major factor in forcing the sixth mass extinction. Catastrophic global temperature increases from pre-industrial levels, based on Intergovernmental Panel on Climate Change (IPCC) estimates, are conservative. For 2023, the IPPC predicted a 1.10 °C increase [1], whereas the actual increase was 1.46 °C [14], and that for 2040 is 1.40 °C [1], with alternative models predicting at least 1.85 °C [14,15]. IPPC underestimates could result from projection models, including a reduction of CO_2_ emissions and increasing CO_2_ capture, and disregarding increased heating through crossing global climate tipping points [14,15]. Meeting CO_2_ emission-reduction targets has failed for most countries, with some countries increasing emissions [16], and prohibitively costly amelioration projections depending on speculative technologies for CO_2_ removal are unlikely to be achieved within realistic timeframes [16,17,18].

Even more alarming are increasing emissions of CO_2_ from disastrous forest fires, rapidly increasing their role as a carbon source rather than a sink [19,20,21], increasing seawater temperatures and acidity, reduced oceanic CO_2_ absorption [22,23], and predictions of the potential collapse by the mid to late 21st century of the Atlantic Meridional Overturning Circulation (AMOC) oceanic current, which affects weather patterns globally, including possible season reversals in highly amphibian-biodiverse major bioregions [24].

Plausibly, more than the current 2062 Endangered or Critically Endangered amphibian species will eventually become extinct in the wild because of catastrophic global heating synergized with other threats [25,26]. Global heating will particularly affect amphibians subject to altitudinal habitat constraints [26,27,28], dependent on permanent stream flows or wetlands [29], or sensitive to forest-habitat destruction through fires [19,20,21,30,31,32] or elevated temperatures beneath tropical forest canopies [33], with predictions that by 2080–2100 up to 35% of anuran habitats will become arid or exposed to worsening drought [34]. Furthermore, global heating will synergize with other environmental stressors, including endocrine-disrupting chemicals [35], micro/nano plastic pollution [36], ice and permafrost melt [37], and the release of toxic chemicals such as endocrine disruptors and heavy metals including mercury and iron [36,38], that will likely be occurring at a much faster rate than can be offset by the slow processes of artificially augmented natural reproduction, resulting in even greater impacts on amphibians and other biodiversity and ecosystems [39]. For example, bird populations have halved in large and otherwise considered pristine Ecuadorian rainforest reserves [40], and insects and other invertebrates have experienced major population declines globally [41]. Because biodiversity is irreplaceable, and its loss so functionally and ethically catastrophic, the precautionary principle directs that we should take the direst global heating predictions as our baseline for an immediate and emphatic response, including the general adoption of reproduction and advanced biotechnologies, biobanking of germplasm and somatic cells, and conservation breeding programs (RBCs) for species perpetuation [42].

However, governments have not taken seriously the needs of intergenerational justice that entitle future generations to a healthy environment [43], sustainable biospheric management that includes the perpetuation of Earth’s biodiversity [3,44,45,46,47], and especially addressing catastrophic global heating [48,49]. Therefore, transformative change and supportive biotechnical, political, and cultural initiatives [50,51] are needed to reduce or prevent biodiversity loss and ameliorate the sixth mass extinction [49,52,53,54].

These transformative approaches must embrace biotechnological advances rather than relying on optimistic stopgap, adversarial, and traditionalist approaches [3,8,50,51,55,56]. Transformative approaches are slowly gaining traction through the International Geosphere–Biosphere Programme [57], the United Nations Convention on Biodiversity [58], the International Union for the Conservation of Nature (IUCN) One Plan Approach to Conservation [59,60], the 2024 Amphibian Conservation Action Plan [61], Amphibian Ark [62,63], and private caregiver conservation breeding programs (CBPs) [3,64,65]. Furthermore, in 2021 the One Conservation concept for species conservation emphatically promoted “reproductive biotechniques” for species conservation [66]. These transformative approaches to varying extents included interventional RBC strategies [3] and the promotion of positive societal changes [67]. Futuristic approaches include species perpetuation through long-term cell and tissue storage in biobanks that, for security, could eventually be secured extra-terrestrially [68]. However, the 2024 Amphibian Conservation Action Plan (ACAP) was not inclusive and disregarded both the enormous potential of private caregiver CBPs [65] and the perpetuation of species through germplasm and somatic-cell biobanking [3].

The application of RBCs offers a transformative change that, irrespective of environmental targets, can: (1) reliably and economically maintain genetic diversity within CBPs (Section 2. [10]) and produce genetically adaptable individuals for repopulation, augmentation, or supplementation programs [3,10]; (2) integrate ex situ and in situ conservation within a broader cultural context, bringing all layers of society as agents of conservation [66,69,70,71,72,73,74,75,76]; and (3) provide the potential for species perpetuation solely in biobanks [3,68,77,78,79,80,81,82,83]. These interventional activities can also benefit humanity through cultural and social inclusion, economic and technological advancement, and educational opportunities [3,67,84]. Overall, RBCs offer a significant advancement in the management of biospheric sustainability and the avoidance of mass extinctions by providing a safety net for the perpetuation of Endangered and Critically Endangered species [3,66,69,70,71,72,73,74,75,76,82,85,86,87]. Sustainability interventions, including amphibian RBCs, have mainly focused on anurans (frogs and toads) and salamanders. Caecilians (Gymnophiona) are also an amphibian order of special concern. Besides a general disregard for caecilians in the public and scientific arena [88], other threats include many data-deficient species of unknown conservation status, a lack of autecological knowledge because of their generally subterranean habitats [89], and little research supporting the development of caecilian RBCs [66,71,90,91,92].

Two decades have passed since the potentials of amphibian reproduction and advanced biotechnologies were first published in Australia in 1999 [70], fertilization with cryopreserved amphibian sperm was developed in Russia [93,94], its use for fertilization in Australia [95,96], and heterocytoplasmic cloning in Japan in 1963 [97,98]. Despite these pioneering achievements, amphibian RBCs have only been implemented for a few species, primarily in wealthy western polities [99,100] and through their satellite conservation programs in the global south [101].

This geopolitical limitation creates a critical gap in amphibian conservation, as very-high-biodiversity countries in the global south are not provided with the necessary independent resources to develop the full potential of RBCs. The development of biobanks representative of all Critically Endangered and Endangered species globally is also particularly lacking [3]. Nevertheless, preventing the immediate extinction of some amphibian species through RBCs has already been achieved. Between 2007 and 2017, there was an increase in amphibian CBPs of ~60%, covering 2.9% of all amphibian species. Half of these CBPs involved repopulation or augmentation, and 70% of these had some success [10,61,102,103,104]. RBCs using sperm biotechnologies have also benefitted the linking of in situ and ex situ conservation programs [99,100]

Cultural factors can play a major role in the widespread adoption of RBCs. Scientific publications and public media should utilize an international, clear, and concise nomenclature system that empowers public engagement and optimizes search-engine visibility [3]. At an international level, this approach, along with oversight beyond the scientific review process, avoids regionalized jargon, misnomers, and euphemisms that hinder the powerful and accurate scientifically based dissemination of information [3,105,106,107]. These initiatives are now even extending to addressing historical injustices in species nomenclature [108]. Pioneering achievements should be properly attributed to promoting global cooperation in a multipolar world (Section 3.7 and Section 4.1).

Philosophical and ethical considerations concerning amphibian animal welfare in RBCs have been particularly prominent with respect to the collection of cells or tissues (Section 5), the release of individuals into potentially hostile ecosystems for repopulation or assisted gene flow (Section 2.2, [103,104]), and advanced reproduction biotechnologies, including assisted evolution or species restoration (Section 4). Despite these ethical considerations (Section 5), intergenerational justice and looming sixth mass extinctions [25,26] demand the prioritization of biobanking of germplasm and somatic cells to enable the opportunist restoration of species through cloning or other advanced techniques (Section 4). Furthermore, to ensure the perpetuation of amphibian species through RBCs, a concerted effort toward international democratic engagement within the emerging multipolar world is essential. This collaboration should broadly include local communities, environmental organizations, and private caregivers’ amphibian collections and their organization’s caregivers [3,64,66,109,110,111,112,113].

In summary, this review explores the potential of RBCs by exemplifying amphibians through (Section 2) management models for genetic diversity; (Section 3) an overview of RBC protocols including their utility, potential, limitations, and research; (Section 4) advanced reproduction biotechnologies, including cloning and assisted evolution; and (Section 5) the ethical and philosophy aspects of RBCs to foster public trust and support. Finally, in Section 6, we summarize current and future applications of amphibian RBCs, particularly the fostering of community engagement and international collaboration to secure a future rich in amphibian biodiversity for the foreseeable future [3]. Overall, this review presents a hopeful outlook for the perpetuation of amphibian biodiversity through embracing current and novel biotechnologies, along with fostering international cooperation through exemplary management and social and cultural sensitivities.

## 2. Genetic Diversity and Species Management

Reduced genetic diversity can lead to genotypes with reduced potential for environmental adaptation, poor health, and lowered fecundity [114,115,116,117,118,119,120,121,122,123,124,125]. A goal of the IUCN’s One Plan Approach to Conservation is the maintenance of sufficient genetic diversity to provide fitness and evolutionary adaptability. It includes all agencies operating in concert to perpetuate species as both wild and captive populations or biobanked genetic resources of individuals [59,60] in a single genetic management unit called a metapopulation [126]. Therefore, a structured approach using genetic databases of both living amphibians and biobanked material combined with genetic modeling is essential for a scientifically credible RBC program [81,82,99,117,119,120,121,122,127].

**Figure 1:**
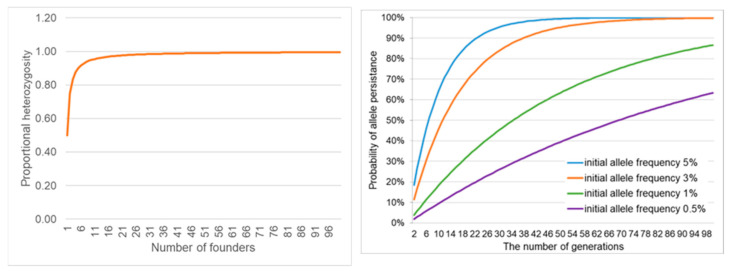
The left panel illustrates the proportional heterozygosity/genetic diversity (*y*-axis) through random sampling of individuals (*x*-axis) for a founding population. The right panel illustrates the probability that an allele will persist in the next generation (*x*-axis) dependent on the number of equally representative reproducing individuals in the captive population (*x*-axis).

Capturing the maximum genetic diversity for ex situ genetic management [81,99,114,115,116,117,128,129] includes the number and proportions of living male, female [120], and biobanked founders [73,76,129]. The IUCN Amphibian Population Management Guidelines recommend 25 founder pairs to represent 99.5% of the target population’s genetic diversity (Figure 1, left panel, [119,120]). An equal number of founder males and females is preferable; however, a bias toward live females would maximize progeny production for release [130].

**Figure 2:**
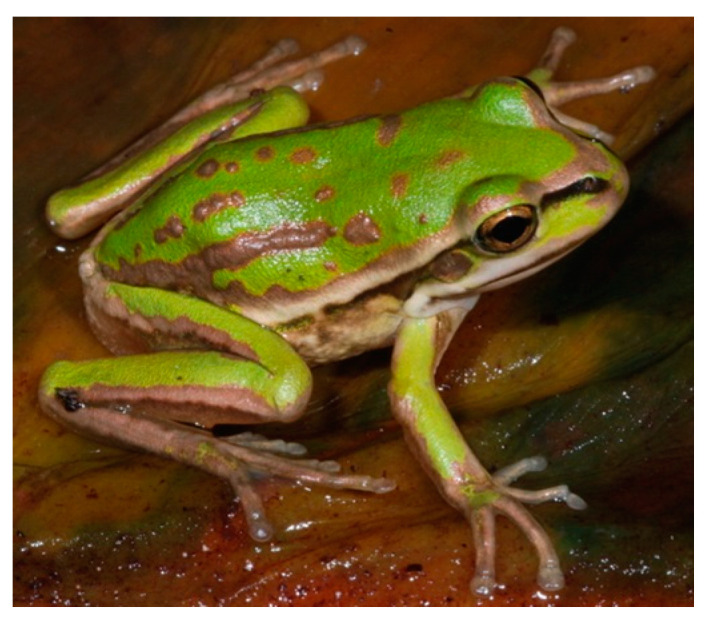
The most common morph of the green and golden bell frog, *Ranoidea (Litoria*) *aurea*, includes both green and gold coloration (image—© 2008 Dr. Peter Janzen).

The number of founders and initial allelic frequency can be used to calculate the likelihood that an allele will persist in future generations (Figure 1, right panel, [92]). However, a greater challenge is the quantification of an allele’s potential for future environmental adaptation. For example, a rare allele might be crucial for developing resistance to a new disease [131] or providing camouflage in a habitat with dramatic seasonality in the chromaticism of vegetation structure [132]. For example, the green and golden bell frog, *Litoria aurea*, a sun-basking species, inhabits wetlands subject to seasonal changes in vegetation color from green to brown, with *L. aurea* varying genotypically and consistently chromatically throughout an individual’s lifespan from pure gold to green, with most individuals being a mixture (Figure 2).

Besides the IUCN Population Management Guidelines [119], alternative approaches have recommended different numbers of biobanked founder individuals and their sex ratios (Table 1, ibid).

Genetic management of mating in CBPs is crucial for preventing inbreeding between closely related individuals [136]. Depending on the species-generation time and lifespan, with an initial population of 25 live males and females, preserving 90% of genetic diversity in a CBP for 25 years requires the maintenance of a minimum number of 100 individuals with strict studbook pairing and 1600 individuals with group management and random mating (Table 2, [120]). Unfortunately, despite careful studbook pairing to track the breeding history of individuals, unexpected deaths, domestic adaptations, and epigenetic effects, can result in the loss of genetic diversity and allelic variation (Figure 1) and progeny poorly adapted to survival in the wild [116,123,124,137].

A key role of the biobanking of germplasm, somatic cells, or tissues is to increase the effective population size beyond the limited number of breeding individuals typically available in CBPs. Sperm storage and artificial fertilization, particularly utilizing banked cryopreserved sperm, are the only current biotechnologies contributing to the prevention of the gradual loss of genetic diversity in amphibian CBPs [71,73,75,76,138]. Biobanked sperm pauses the genetic aging process within a CBP and provides a reservoir of genetic variation, including rare alleles that might be crucial for future adaptation to environmental changes such as disease resistance [131], modified ecosystems [4,6], or predators [132].

**Figure 3:**
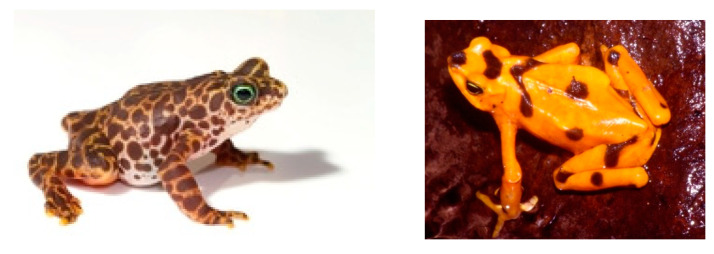
Left, *A. certus* (Image—© 2019 Brian Gratwicke, Creative Commons Attribution 3.0 (CC BY 3.0)); right, *A. zeteki* (Image—© Peter Janzen).

The benefits of biobanking sperm are exemplified by real-world examples. Case studies involving three frog species demonstrated how using biobanked sperm for breeding (back-crossing) reduces the required size of the CBP’s population, minimizes inbreeding, and lowers costs when compared to maintaining large numbers of individuals [139,140]. Biobanked sperm can restore genetic diversity to highly inbred color morphs, irrespective of their genetic diversity, in private caregiver collections, thus avoiding costly intuitional CBPs altogether and encouraging private caregivers’ engagement in species perpetuation. For instance, the popular, attractive, and highly endangered *Atelopus* sp. (Figure 3) could potentially be perpetuated through private caregiver collections along with genetic diversity provided through cryopreserved sperm [64,141]).

Some publications have recommended the biobanking of germplasm and, to a lesser extent somatic cells, of all threatened amphibian species, Vulnerable, Endangered, and Critically Endangered [128,133]. However, because of limited resources, biobanking should primarily focus on the immediate needs of the current ~1630 Endangered and particularly Critically Endangered species [9,10,11]. Vulnerable species are not in immediate danger of extinction [9,11,142], and their management should emphasize autecological IUCN recommendations for fieldwork, research and monitoring, and particularly habitat protection, including encouraging community engagement [3,9,11,142]. Nevertheless, if resources are available, opportunistic biobanking of some Vulnerable species close to endangerment could be undertaken before the possible loss of genetic diversity with major population declines occurs.

### 2.1. Genetic Bias in Founder Sperm Collection

Minimizing anthropocentric, terminal-investment effect and founder selection bias, ensures the optimal representation of the target population’s genetic diversity [75,76]. Anthropogenic bias occurs through collectors favoring traits such as vocalization [143], antagonism and larger size [144,145], and chromaticism [146]. However, these traits may not represent a species’ adaptive genetic diversity [147,148,149], as they are weakly associated with mating systems, habitat types, and life history [145]. Although age is associated with size and senescence theory predicts loss of sperm quality, and therefore yield, no effect was found by Watt et al. [150].

Evolutionary trait constraints are possible through increased antagonistic inter-male competitive traits [144,145] corresponding to decreased female mate choice, along with ‘sneaker’ males and delayed partial-clutch fertilization influencing the success of fertilization [151]. Further research is needed to fully reveal the relationships between sperm genetic diversity [152,153], female sperm choice in fertilization [154], and the fitness of resultant progeny [155].

Biases could also be due to genetically predisposed characteristics toward pathogens, including susceptibility to more overt parasite-mediated behavior [156], greater size corresponding to the intensity of infection [157], or through terminal investment effects where sick or potentially dying individuals invest larger resources than usual to increase reproductive output [158]. Terminal investment effect was evident through increased spermatogenesis of males from two anuran families during hormonal stimulation when infected by the major-threat fungal pathogen of amphibians [158]. Furthermore, infections with parasites [157] from taxonomic groups with Endangered species [159,160,161] also increased spermatogenesis in one species [158,160]. However, in another species, male advertisement and mating outcomes were lower in fungal-pathogen-affected males [162]. Besides potential bias toward parasites, sperm collection through hormonal stimulation includes the possibility of lower sperm yield from less-mature or more stress-prone males (See Section 4.2. [163]).

### 2.2. Assisted Gene Flow

Assisted gene flow (AGF), where desirable genotypes are transferred between CBPs, biobanks, and wild populations to reduce inbreeding depression, is an emerging RBC that could potentially increase population fitness but also entails considerable risks [10,75,99,102,164,165,166,167]. Reduced inbreeding depression should increase reproductive capacity, evolutionary adaptability, and consequently, species survivability [114,115,116,117,118,119,120,121,122,123,124,128,142,168,169,170]. However, these advantages are dependent on the target population’s size, its unique genetic diversity, and whether it is a metapopulation, including its fragmented sub-populations, or a genetically unique population isolated over geological periods [117,171,172]. AGF strategies toward fragmented subpopulations should utilize pooled genetic diversity from the core population [173] to avoid the diminished genetic diversity of the most divergent or fragmented populations [169,174]. In contrast, AGF toward isolated populations should be avoided [175], except where historic bottlenecks have reduced genetic diversity to the extent of demonstrably reducing fitness and survivability [176,177]. Any advantages of AGF also depend on natural selective pressures toward genetic diversity that favor survival in the wild, irrespective of any loss of genetic diversity [114,117,171,172].

Assisted gene flow toward wild populations can include any life stage; however, various beneficial genetic traits manifest throughout a species’ life history [178], for instance, fungal pathogen lethality varies between the tadpole and the adult stage [179]. Natural selection from early stages favors the retention of endemic genes or beneficial AGF genes along with the loss of detrimental genes [180,181,182,183,184,185]. Therefore, effective AGF strategies toward advantageous genotypes are through egg masses or early larvae. Oocytes could be sourced through on-site collection from wild gravid females, or females in CBPs, and fertilized with genetically diverse sperm [99].

Small, intermittently fragmented wild populations are targeted for AGF programs [186], with the potential benefits and risks being dependent on the population’s most recent fragmentation and any subsequent gene flow. However, the genetics of naturally fragmented populations are challenging to profile and are likely naturally trending toward inbreeding rather than outbreeding [187]. Therefore, AGF could reduce both fragmented and isolated populations’ survival through outbreeding depression and loss of alleles [187,188], the introduction of harmful genes [164,187], influencing male/female incompatibility [189,190], and pathogen transmission [122,123,124,142].

Moreover, the impacts of inbreeding depression and the loss of genetic diversity on the extinction risk of amphibian populations, although widely theorized, are not evidenced in amphibians’ or other taxa’s declines or extinctions through reduced genetic diversity [191], and many species thrive with very low genetic diversity [192,193]. These include island endemic species with effective population sizes of 500–1000 individuals [193], a population size close to the theoretical minimum required to maintain genetic diversity [177]. An increasing number of species of amphibians [10,61] and other taxa [194] are also being repopulated in the wild from a few founders. These cases offer optimism for the survival of small populations of amphibians if a suitable habitat remains for their survival without the potential risks of AGF-supplemented genetic diversity [179,181,195].

Some large-scale repopulation programs are based on the hope that individuals develop unique genotypes that ameliorate or counteract new ecological realities such as lethal exotic pathogens [196]. However, these programs for toads [103,197] and frogs [104] have yet to result in viable populations. Nevertheless, some initially very small natural populations persist despite the prevalence of lethal pathogens [104,179,198]; however, whether this is due to new genotypes, habitat preference, or other behavior at different life stages is uncertain [179,198]. Other programs simply bolster populations through releases, with a recent emphasis on increasing AGF [77].

The potential for outbreeding depression [187] and pathogen transmission [199,200,201] should be evidenced before implementing AGF, and there should be genetic and demographic monitoring both pre- and post-AGF [202,203]. Genetic and demographic monitoring will be a costly process over prolonged periods [177]. A major consideration before implementing costly and potentially risky AGF is that habitat loss may be the overwhelming cause of a species decline. In these cases, habitat protection, amelioration, or provision could maintain species for the time being without the risks and costs of AGF [181,193,195,204], with survival in the wild proving environmental adaptation and genetic fitness [194].

In summary, the benefits of AGF depend mainly on the genetic diversity of the target population, the translocated genotypes, the target population size, proportionate release numbers of individuals with highly beneficial genotypes, the life stage at release, and environmental selection toward the translocated genotypes. The repopulation of captive-bred individuals into the wild can also pose significant risks through outbreeding depression and pathogen transmission. Thorough planning of AGF is essential, including disease risk assessments, genetic management strategies, and post-release monitoring of demographics and population genetics including population viability analysis and computer modeling.

## 3. Reproduction Biotechnologies

Amphibian reproduction modes correlate with those of fishes, and amphibian RBCs have reciprocal practical applications in fish reproductive management, biobanking of germplasm, and CBPs [127,205,206,207,208,209,210]. Here, we provide an overview of amphibian RBCs, including protocols, their application, and future directions, along with the practicalities and ethics of sample collection, and address some recent historical and technical misrepresentations (also see Section 5). Amphibian sperm and oocyte collection, refrigeration of sperm at 4 °C or cryopreserved storage, and artificial fertilization require basic laboratory facilities and animal-handling procedures [211], whereas advanced techniques for cell culture and restoration technologies, such as cloning and assisted evolution, require sophisticated laboratory facilities and technical expertise [82]. Webinars are available online describing details of amphibian reproduction biotechnologies for the hormonal stimulation and collection of sperm and oocytes and their use for in vitro fertilization [212].

### 3.1. Life Stages and Sample Collection

A generalized caution against using early life stages to provide adults for gamete collection was recently published [99]. However, the males of many anuran species mature in less than one year, and both males and females of most species in two years or less [213,214], with salamanders having longer maturation periods than anurans [71,215]. Average oocyte numbers show that many Endangered and Critically Endangered anuran species in the wild can provide surplus oocytes, larvae, and early juveniles [26]. In any case, only a few oocytes are needed for biobanking embryonic stem cells. Different life stages of many Endangered and Critically Endangered species could also be sourced from zoos, CBPs, and private caregiver collections [61,64].

**Figure 4:**
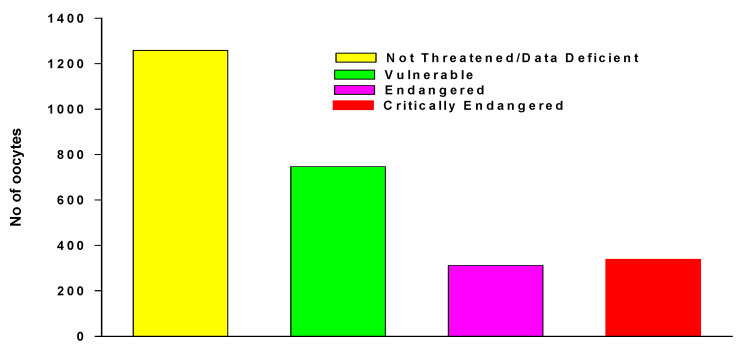
The average anuran clutch size by IUCN Red List endangerment status.

We accessed anuran oocyte numbers in Guirguis et al. [26] taken from IUCN assessments of clutch sizes of 1611 anuran species in total, with 405 threatened species. We added new data from 178 non-threatened anuran species, and then categorized the combined data, using IUCN Red List criteria (Figure 4).

**Figure 5:**
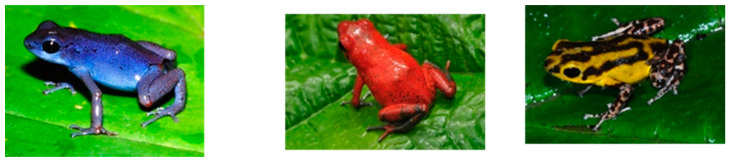
Left, blue morph; middle, red morph; right, yellow morph. Different color morphs of the strawberry poison dart frog, *Oophaga pumilio*, and many other amphibian species are very popular with private caregivers and have a long history of domestication (Images Peter Janzen).

Clutch size declined with increased Red List threatened species status, but not between Endangered and Critically Endangered species, and was highly species-specific. For example, *Atelopus* includes a very high proportion of Endangered and Critically Endangered species [216] and spawns hundreds of oocytes [26]. In contrast, similarly highly threatened *Oophaga* species lay individual eggs [216,217,218]). Both *Atelopus* and *Oophaga* species successfully reproduce in captivity and many colour morphs are held in private caregiver collections (Figure 5, [64]). However, although 96% of *Atelopus* species’ habits are protected, declines and extinctions are continuing [216], and management plans do not emphasize the need for biobanking and disregard the potential of private caregiver collections [217,218]. Nevertheless, a nascent RBC program for *Atelopus* is underway in Ecuador [141].

### 3.2. Vouchering

Vouchering, the process of collecting and preserving physical specimens, can opportunistically provide valuable authenticated sources of biobanked sperm, and other cells or tissues, for RCBs [219,220]. Other conservation benefits of vouchering are taxonomic distinction of metapopulations [126] from genetically divergent isolated populations [204] as distinct management units [221,222,223], informing the epidemiology of amphibian extinctions and declines [196], and providing life history and ecological information required to facilitate CBPs and release programs [142]. Unfortunately, a decline in vouchering has impoverished museum collections that cannot adequately address the looming problem of mass extinctions driven by global heating and other causes [222]. Zoos are making significant contributions to biobanking and vouchering [223], and these contributions could be more widely adopted throughout RBCs, veterinary services, and animal welfare centers.

### 3.3. Reproduction Biotechnologies, Gamete Collection, Donor Stress, and Pathogens

The least stressful reproduction biotechnology is the simulation of natural environmental cues to promote mating and spawning in anurans [71,224] and salamanders [71,215,224]. Cues of temperature and humidity are generally intermittently circannual in terrestrial temperate anurans and continuous in terrestrial salamanders and tropical anurans [225,226], with precipitation generally triggering reproduction [227]. Environmental cues are used by private caregivers for most species to produce large numbers of progeny [64] and in augmentation or repopulation programs [215,224]. Temperature-regulated brumation can also promote gonad maturation, mating, and spawning responses to hormonal stimulation [74,224,228], with males and females of at least one salamander requiring different temperatures to optimize reproductive maturity [229]. Sex differences in response to environmental and social breeding cues are found in one anuran [230].

Amphibian progeny can also be produced through in vitro fertilization with fresh sperm or sperm refrigerated for days to weeks or cryopreserved (Section 3.4, Section 3.5 and Section 3.6). Sperm from the testicular tissues of euthanized males has contributed to foundational [76,93,94,95,231] and recent amphibian RBCs [86,189,232,233,234] and produces the high sperm numbers needed for the efficient production of large numbers of individuals for repopulation, augmentation, and gene-flow programs.

The IUCN, ACAP 2024, mandates that “*In cases where gamete recovery is part of a conservation strategy euthanasia is not recommended*” [61] (p. 294). However, sperm collection from testicular tissue is the preferred method if males are available [71,74,76,86,95,96,231,232,233], as it only requires euthanasia through injection and no further live handling [86,95,96,232,233]. With injection and euthanasia, the dissection of testes and the production of sperm suspensions are achieved in a few minutes [189]. Sperm collection from testicular tissue is preferable (1) when males can be taken from the wild without threatening populations, (2) with surplus males from CBPs [76,86,232,233], (3) with vouchered males (Section 3.1), (4) for the collection of large, high-quality sperm yields from small and especially very small amphibians [71,105], (5) with mortalities after strict pathogen screening [200,201], (6) for the testing of multiple female/male compatibilities [189], and (7) to produce quantities of concentrated sperm suspensions of consistent pH and osmolarity [189]. Sperm collection from testicular tissue is essential for species recalcitrant to hormonal stimulation [10,235].

**Figure 6:**
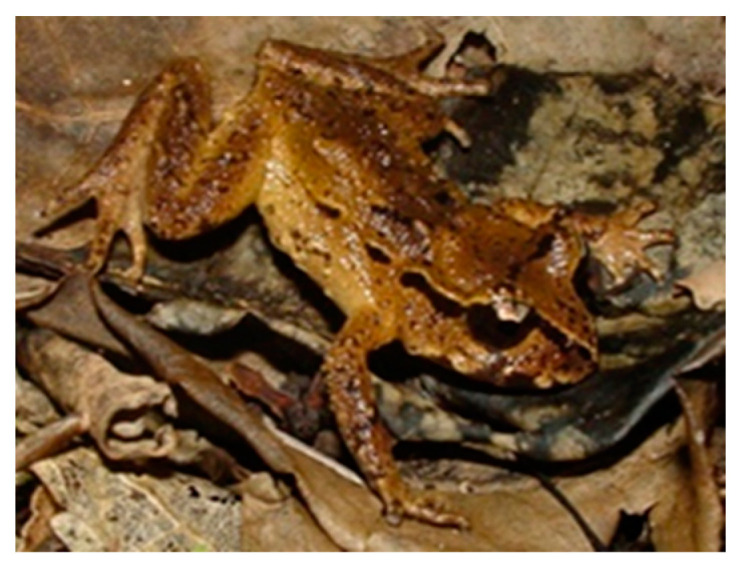
Hamilton’s frog (*Leiopelma hamiltoni*), where hormone stimulation produced extremely low sperm concentrations of poor-quality sperm. Image by Phil Bishop. Attribution ShareAlike 2.5. https://en.wikipedia.org/wiki/Hamilton%27s_frog#/media/File:Leiopelma_hamiltoni02.jpg (accessed 19 September 2024) Phil Bishop, CC BY-SA 2.5 https://creativecommons.org/licenses/by-sa/2.5, (accessed 19 September 2024) Wikimedia Commons.

Hormonal stimulation (misnamed hormone therapy in some amphibian RBC literature [236]) generally requires injection, as with collection from testicular tissue, but does not require euthanasia. Hormonal stimulation has been published for more than 40 species and applied for propagation for release in some species [61,75]. Unfortunately, many studies present sperm yields as concentrations but not sperm numbers, making it difficult to assess the utility of this technique [61,75]. Nevertheless, the utility of hormonal stimulation for many species is limited by generally low concentrations of inferior-quality sperm [10,235]. For instance, hormonal stimulation produced extremely low sperm concentrations and poor-quality sperm with the Endangered frog *Leiopelma hamiltoni* in the basal Anura family Leiopelmatidae, (Figure 6, [235]). A similar response is also likely with *Le. archeyi*, the highest-priority amphibian species on the Zoological Society of London’s Evolutionary Distinct and Globally Endangered (EDGE) list [206]. In contrast, exceptionally large quantities of hormonally stimulated giant salamander semen are readily stripped for *Andrias* (giant salamander) aquaculture in the People’s Republic of China, and in the USA for the development of RBCs for *Cryptobranchus alleganiensis* [71,215,237]. Nevertheless, even if not generally practicable for large-scale progeny production, hormonal stimulation can yield sperm quantities, whether motile or immotile, that are suitable for intracytoplasmic sperm injection (ICSI) [238,239,240,241,242], and low sperm numbers can produce larvae that are then raised to reproductive maturity to produce large numbers of progeny for release [3].

After hormonal stimulation, some anuran species will spontaneously express spermous urine after abdominal massage [243,244,245], or, to sample spermous urine, a cannula or a pipette tip can be inserted into the urethra [10,246]. Salamander sperm can be collected after hormonal stimulation in pre-spermatophores or in gelatinous fluid [247,248], spermous urine [247,248], semen collected by massaging the lower oviduct and cloaca [215,247,249,250], or through the natural deposition of spermatophores [76]. However, hormonal stimulation using injection is stressful to small, delicate salamanders [249], including the highly threatened plethodontids that comprise 65% of salamander species [71,249]. Alternative methods for hormonal stimulation of spermiation in small, delicate salamanders or anurans include topical or nasal application [71,251,252]. The more robust body shape and, consequently, lower skin-surface to body-mass ratio could make small anurans less amenable to the topical or nasal application of hormones than salamanders of the same body weight. On the other hand, anurans have the advantage of a patch of skin in the ventral pelvic region that is permeable to aqueous solutions of hormones [253]. Spermiation was stimulated using a novel technique of hormonally injected crickets fed to salamanders, termed non-invasive oral bioencapsulation. This technique avoids any stress of hormone stimulation for both large and small salamanders [254].

Females of both anurans and salamanders are generally more recalcitrant to hormonal stimulation for oocyte collection than males for spermiation and often require priming doses [74,243,247,255,256,257]. Mature oocytes can then be collected through ovarian excision [95], abdominal massage, or cannulation [71,72,73,74,247,258,259]. Improved targeting of hormonal stimulation has been achieved through ultrasound by identifying mature ovaries in both anurans [260] and salamanders [244,247]. Ovarian excision and spawning into physiological saline (Section 3.6) are low-stress techniques for the sampling of large numbers of oocytes [243,257]. Unseasonal hormonal stimulation of oocytes did not affect oocyte quality in one cool-temperate species [261].

An important factor in selecting gamete collection methods is pathogen transmission [200,201]. Testicular sperm may be contaminated by pathogens such as internal parasites and viruses [157,159], whereas hormonally stimulated sperm in various forms may be additionally contaminated by bacteria, fungi, and external parasites [237].

### 3.4. Sperm Motility and Integrity

Comparative evaluation of spermatological techniques in methods and terminology are confounded by inconsistencies in the evaluation of metrics including motility, membrane integrity, and DNA integrity [75,76,150,205,250,262,263,264,265,266,267]. Basic metrics of sperm motility are the percentages of immotile sperm, activated sperm as non-swimming motion, swimming sperm, and sperm speed or velocity. Sperm motility in amphibians is activated by a lowering of osmolarity from that found in plasma, and higher osmolarities than in nature to extend the period of motility are frequently found in RBC research ([76,234], Section 3.8). The period of motility of anuran sperm is also important during in vitro fertilization and increases as osmolarity rises above that of pond water until the activation osmolarity is reached, although the percentage swimming and velocity of sperm declines at higher osmolarities [76]. Besides various physiological salines where sperm motility is dependent on osmolarity, compounds to stimulate or extend motility such as ATP/adenosine monophosphate [76] or phosphodiesterase inhibitors [234] have failed. Capacitation of anuran sperm and that of salamanders occurs in the oocyte gel [268] and has not been recorded for caecilians [66].

Sperm motility is subjectively assessed by eye [95]; however, meaningful comparative studies need specific setups and methodologies of computer-aided sperm assessment (CASA) to give exact percentages and speeds of swimming sperm [269], along with other motility vectors for more precise analysis [76,270]. Damage to sperm morphology assessed by observation or vital stains includes the integrity of acrosomes [186] and mitochondrial collars or sheaths [266,271,272] (misnamed in some of the amphibian RBC literature as “mitochondrial vesicles” [271,273]). DNA integrity is assessed through comet assays and other tests [228,264,265], with other biochemical tests sometimes used for amphibians [262].

However, although sperm from testicular tissues reliably achieves fertilization, the rate compared to sperm from spermous urine can vary in proportionality from high to low and may depend on reproductive maturity and be species-specific, and little difference was shown between the cryoresistance of sperm from spermous urine or sperm from testicular tissues ([274], Section 3.8). These factors should be considered in comparisons of sperm motility and quality between sperm from spermous urine and from testicular tissue, and further research should be undertaken to provide more meaningful comparisons (Section 3.8).

### 3.5. Refrigerated Storage of Sperm

Refrigerated storage at 0–4 °C is a useful technique for the short-term storage of amphibian sperm. Refrigerated storage enables delayed in vitro fertilization when there is asynchrony between sperm collection and oocyte availability [262] or for transport between breeding groups [99]. Refrigerated storage for ~10 min can acclimate sperm to cryoprotectants [266] and be used to delay cryopreservation for days to weeks [76,96]. Refrigerated storage of anuran sperm is highly successful in cadavers [228,245], whole testes [96], macerated testicular tissue [96], and spermous urine [75]. However, refrigerated salamander sperm only remains motile for a day to several days [71,76] or for weeks when held in spermatophores [275]. Oxygenation [276,277] and antibiotics to reduce bacterial concentrations improve sperm storage periods while maintaining sperm concentrations, with antibiotics reducing the chance of pathogen dissemination [75,276,277,278,279,280]. Refrigerated sperm may be inactivated in hypertonic solutions, in testes, or in testicular macerates, with motility minimized at low temperatures in spermous urine [245,278,280,281,282].

### 3.6. Sperm Cryopreservation and Freeze Drying

Cryopreservation is a freezing process that protects sperm using special solutions to create sperm cryosuspension and tailored freezing rates. The cryopreserved sperm can then be held indefinitely in biobanks [75,76,283]. Sperm cryopreservation uses aqueous formulations, termed cryodiluents, mixed with sperm from testicular tissues or spermous urine to produce cryosuspensions that are then frozen [75,76]. Cryodiluents are formulated from sperm- penetrating cryoprotectants, such as dimethyl sulfoxide (DMSO, [93,94] or dimethylformamide (DMFA, [228,266]), and non-penetrating saccharides or salts, with supplements such as buffers, fetal bovine serum, and antibiotics [75,76]. Fertilization was first achieved with cryopreserved anuran testicular sperm in 1996 using DMSO [93] and with hormonally stimulated sperm in 2011 using DMFA, then a novel cryoprotectant to amphibians [266]). Mouse sperm has also been freeze-dried and then stored at room temperature [284] and remained viable, but this technique has not been tested in amphibians, either for fertilization [71,76] or for ICSI [238,239,240,241]

A broad canvas of the RBC community in 2019 recommended 5–10% (*v*/*v*) DMSO or DMFA and 1–10% (*w*/*v*) saccharide as cryoprotectants for amphibian sperm and the use of slow to moderate cooling rates [76]. Nevertheless, ranges of 12–15% DMSO have proved successful with anurans [93,94,96,228,232,233,266], with 12% DMFA proving superior to DMSO in a pioneering study with urinal sperm [266] and then generally used in other studies of hormonally induced sperm [270,274,283]. Both fast and slow cooling rates have recently been successful [75,76,270]. Cryosuspension osmolarity has shown a significant effect on cryopreservation in some studies [232], while other studies have shown little effect [274]. The freezing of sperm over a range of freezing rates can be achieved by constructing a simple and inexpensive device first used for fish sperm [285], with cooling rate impacts on amphibian sperm found in [270]. The broad differences in successful cryopreservation protocols, even within the same species, may be due to subtle variations in techniques for cryoprotectant penetration, freezing regimes, the thawing and washing of cryoprotectants from cells [75,76], seasonal effects on sperm quality [281], in vitro fertilization techniques lacking meaningful baselines (see Section 3.8), unconcise sperm-quality metrics (Section 3.4), and sperm genotypic and phenotypic characteristics [125,283]. There was little difference between the cryoresistance of urinal sperm or sperm from testicular tissues in *Bufo bufo* [274].

### 3.7. Oocyte Storage

The storage of oocytes enables delayed in vitro fertilization if there is asynchrony between the availability of oocytes and sperm [75,76,286]. Because of their high yolk content and large size, the cryopreservation of anuran oocytes is not yet practicable [287]. The viability period of unfrozen oocytes during storage depends on a species’ natural spawning temperature, the storage temperature, and the oocyte’s osmotic, ionic, and gaseous environment [74,96,231,282,288,289]. Early conventions for oocyte storage assumed that ionic formulations slowed oocyte gel hydration (described as hardening), which blocked sperm penetration [158,288,289]; however, research over a broader range of species showed that the hydrated oocytes of some species are fertilizable for an hour or more [231]. Unhydrated and refrigerated ovarian oocytes and post-ovarian oocytes of cool-temperate species have remained viable for many days [282], whereas a pressurized gaseous environment extended storage life further [290].

Oocyte storage is highly dependent on the target species’ natural spawning temperature, where the oocytes of species spawning in cold water may remain viable for many days [282], and tropical or subtropical species only for hours [96,288,289]. Studies with a single species showed that a percentage of post-ovarian oocytes undergo a very rapid loss of fertility [189], possibly a female sperm choice mechanism for greater progeny heterozygosity with polyandrous species [291], or to maintain sub-population environmental adaptability where female/male genetic incompatibility lowers fertilization rates in anurans with little dispersal ability [189,190]. In other species, some oocytes resist fertilization during spawning, leaving fertilizable oocytes for later fertilization [151]. However, the physiological or genetic mechanisms behind selective oocyte fertility loss over time, and any subsequent genetic biases toward progeny, are undetermined. Therefore, besides species-specific temperature effects, the mechanisms for the loss of oocyte viability in aqueous solutions generally appear to be through the diffusion of ions or proteins from the oocyte gel needed for sperm motility or for oocyte metabolism [292,293,294,295] rather than gel hydration [288,289]. Specific genotypic mechanisms also influence the storage period and even extend to individual oocytes [189].

**Figure 7:**
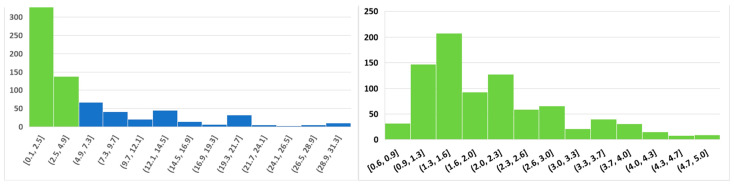
The *x*-axis shows the number of anuran species, and the *y*-axis shows oocyte volumes (mm3). Left panel, 805 species with oocyte diameters up to 4.9 mm and volumes up to 31.3 mm^3^. Right panel, ~430 of the 805 species from the green bars in the left figure with oocyte diameters ≤ 1.8 mm and volumes ≤ 5.0 mm^3^.

The cryopreservation of amphibian oocytes or early embryos has no parallel with techniques used for mammalian oocytes, which are very small, ~0.08–0.20 mm in diameter, and have minimal yolk content [296]. However, the cryopreservation of amphibian oocytes or embryos includes many parallels with fishes [207,208,287] due to both possessing highly fatty and structured egg-yolk and oocyte sizes. Nevertheless, the success of cryopreservation techniques for fish embryos up to 0.8 mm in diameter Ref. [207] is challenging to apply to the large size of more than 95% of amphibian oocytes that are over 0.9 mm in diameter (Figure 7). Therefore, currently, the most promising techniques to perpetuate the amphibian female genome is using cryopreserved biomaterial for heterocytoplasmic cloning (Table 3, ibid) or stem cells to generate ovaries in surrogate species (Section 5, [287]).

### 3.8. Fertilization; Sperm Concentrations, Fertilization Periods, and Rates

The critical endpoint of fertilization rates when comparing sperm concentrations and quality is confounded by varying techniques and terminologies, even with respect to misinformation in historic attributions. The term “fertilization” is generic and includes in vitro (traditionally also “artificial fertilization”) fertilization, where sperm is placed over oocytes, as presented in [189,286,308], but also artificial insemination with sperm placed internally [309] and intracytoplasmic sperm injection (ICSI) with sperm placed into an oocyte [238,239,240,241]. In vitro fertilization is the critical endpoint of most amphibian BBC research and has been achieved with scores of anuran species and tens of salamander species [61], but artificial insemination has been trialed with only one salamander species [309].

The first book devoted to amphibian BBCs published in 2022, “*Historical Perspectives on the Development of Amphibian Reproductive Technologies for Conservation*” ([286], p. 1), stated that in vitro fertilization in amphibian RBCs was “dry fertilization” and attributed “dry fertilization” as a pioneering experimental achievement to Rugh, 1961, in the USA [310]. Historically, dry fertilization was pioneered in 1856 in Russia, where inactivated fish sperm was mixed with oocytes, and then, after a short period, the sperm was activated with water to achieve fertilization [311]. In contrast, Rugh first published the placement of activated sperm on anuran oocytes in 1934 [312]. This technique was then furthered in other early studies [288,289], first used in amphibian RBCs in Russia in 1996 [94], and subsequently generalized in amphibian RBCs [228,243,244,245,257].

**Figure 8:**
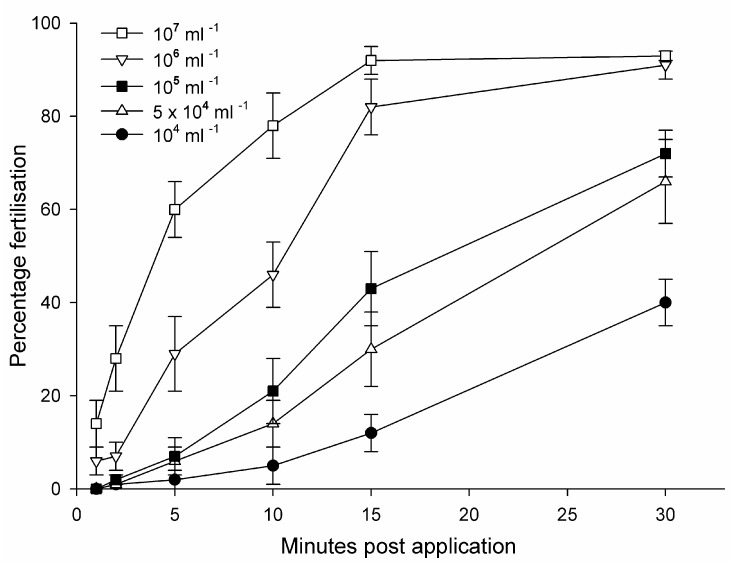
*Rhinella* (*Bufo*) *arenarum* (Bufonidae) fresh testicular sperm concentrations, the percentage of in vitro fertilization, and minutes post application before washing with fresh water [314].

Our knowledge of artificially collected sperms role in in vitro fertilization is limited by the literature lacking comparable sperm-quality metrics (Section 3.4 [313,314]), sperm concentrations and fertilization periods [95,231,266,314,315], sperm activation and fertility in different osmotic environments [75,76,234,250,315,316], and species- and maturation-specific differences between sperm from testicular tissue and from spermous urine ([264], Section 3.4). The effect of osmolarity can even extend to the osmotic environment of donor males [316]. Fertilization curves are the endpoint of sperm quality; however, few publications have included amphibian fertilization curves, which were first presented in the general scientific literature in 1975 [314] and the RBC literature in 1998 [96]. Furthermore, the fertilization period in most amphibian studies is 10 min and refers to concentrated sperm suspensions at high osmolarities [96]. However, comprehensive fertilization curves at different sperm concentrations show that fertilization rates with *Rhinella* (*Bufo*) *arenarum* increased for at least 30 min post application, with the post-application period being more influential than linear sperm concentrations on fertilization rates (Figure 8, [314]).

Other fertilization curves show saturated fertilization with sperm from testicular tissue at concentrations of 2.5 × 10^5^ mL^−1^ after 60 min [315], ~10^4^ mL^−1^ after 60 min [231], ~10^6^ mL^−1^ after 10 min [96]), and 10^5–7^ mL^−1^ after 60 min [289]. The fertilization rate, in general, will also depend on the relatively short motility period and lower quality of cryopreserved sperm compared with fresh sperm [317,318]. For instance, after 15 min application, *Rana temporaria* spermous urine at ~10^6^ mL^−1^ provided ~45% fertilization, while testicular sperm provided only ~2% fertilization. Spermous urine achieved saturated fertilization at ~10^7^ mL^−1^ but testicular sperm only at ~10^8^ mL^−1^ [266].

The need for robust fertilization curves to compare fertilization rates was exemplified through much higher fertilization rates than expected with cryopreserved sperm from the spermous urine of *Anaxyrus* (*Bufo*) *fowleri*. Control fresh swimming sperm provided ~85% saturated fertilization at ~4.2 × 10^6^ mL^−1^ concentration, whereas after cryopreservation, swimming sperm at only ~2% concentration of the control provided ~20% fertilization [319], even though the fertility of cryopreserved sperm is lower than fresh sperm [266,274]. A robust fertilization curve could explain this apparent enigma and, in general, provide baselines for meaningful comparisons among all studies.

## 4. Advanced Reproduction Biotechnologies (aARBs)

Advanced Reproduction Biotechnologies (aARBs) can perpetuate species through the cryopreservation of cultured or uncultured somatic cells [320] with subsequent cloning [99,208,287,296,321,322], or stem cells used for the generation of gametes in surrogate species [77,80,81,287]. The generation of gametes includes the implantation of cryopreserved immature ovarian follicles, primordial germ cells, or induced pluripotent stem cells to generate chimeras [73,77,287]. These techniques were first introduced to the amphibian RBC literature in 1999 [70].

### 4.1. Cloning

Heterocytoplasmic cloning enables species restoration solely from biobanked nuclei through surrogate species [99,208,287,296,321,322], and the critical need to develop heterocytoplasmic cloning has not received the attention it deserves in amphibian RBCs. To perpetuate amphibian biodiversity, heterocytoplasmic clones must develop to reproductively mature and fertile females to produce oocytes for fertilization with cryopreserved sperm. Homocytoplasmic anuran clones from embryonic cells were developed to late blastula as early as 1952 [297], with heterocytoplasmic cloning first accomplished with anurans in Japan and with salamanders in France (Table 3, ibid). Heterocytoplasmic clones were then developed to the gastrula stage in 1957 [298], early neurula in 1958 [299], adults in 1961 [300], with naturally mating and spawning adults producing viable offspring in 1963 [97,98,301], and second and third generations from 1963 to 1972 [302,303], and from as early as 1971/1972, adult heterocytoplasmic clones were produced from a wide range of other anurans [304,305] and also from salamanders (*Pleurodeles* sp.) (Table 3, ibid; [306,307]. In 1998, nuclei from cryopreserved totipotent cells were developed to the gastrula stage [322] using the techniques of [321]. These foundational studies and the extraordinary rate of the development of de-extinction (species restoration) projects for other taxa relying on cloning [323] herald a new age in amphibian cloning that will lead to species perpetuation solely from cryopreserved nuclei. This will greatly reduce the need for CBPs for species unable to survive in the wild or CBPs for species of little cultural interest. This advance will not only relieve some of the financial burden through maintaining live amphibians but will also have considerable animal welfare benefits.

The first book devoted to amphibian RBCs published in 2022, “*Historical Perspectives on the Development of Amphibian Reproductive Technologies for Conservation*” ([286], p. 1), attributed “*one of the most noteworthy and groundbreaking accomplishments toward amphibian reproductive technologies*” to Gurdon’s 1962 publication of amphibian homocytoplasmic cloning in the USA [324]; this accomplishment also resulted Gurdon receiving a Noble Prize in 2012 [325]. However, Gurdon’s homocytoplasmic cloning was not pioneering and extended Briggs and King’s 1962 homocytoplasmic cloning of amphibians in 1952 [297], and neither concerned heterocytoplasmic cloning a technique essential for amphibian species perpetuation [286]. Furthermore, the 2012 Nobel Prize for Physiology or Medicine was awarded jointly between John Gurdon and Shinya Yamanaka [325], not for cloning, but for the reprogramming of nuclei to pluripotency [325], as discussed by Gurdon in [326] his acceptance speech “The Egg and the Nucleus: A Battle for Supremacy” [327]. Reprogramming of nuclei to pluripotency was not used in the heterocytoplasmic cloning listed in Table 3, as pluripotent nuclei are readily available from early amphibian embryos (Table 3, ibid).

### 4.2. Assisted Evolution

Traditional methods of assisted evolution include the selection of desirable traits in domestic species [328], as found in amphibians with private caregivers’ colored morphs [64]. Other assisted-evolution techniques for amphibians include the natural selection of beneficial traits through mass releases [103,104] and approaches toward genetic engineering to provide pathogen immunity or adaptability to the climate crisis [61,129,329,330]. In contrast to most amphibians, the benefits of assisted evolution in mammals are challenged by their low reproductive rates [82]. Nevertheless, assisted evolution is a crucial strategy to select or increase amphibian genetic diversity or for species restoration [3,61,77].

## 5. Ethics and Communication

By addressing ethical principles, communication challenges, and cultural normalization, we can fully realize the potential of RBCs [3,44,45,46]. The ultimate standard for the ethical treatment of all animals is “*The physical and psychological well-being of an animal* (sic, in captivity)*. It is good or high if the individual is fit*, *healthy*, *free to express natural behavior*, *free from suffering*, *and in a state of wellbeing*.” [331].

Ethical principles dictate that RBC practitioners are moral agents responsible for the well-being of their moral subjects, the species of concern [332,333]. Practitioners’ ethical motivation toward amphibian RBCs is driven by a responsibility to perpetuate amphibian biodiversity while avoiding unnecessary harm [61]. Practitioners are guided by the precept, “*How would I like it if I were them?*”, an ethical principal present in all cultures with ethical traditions [333]. RBC animal-ethics standards include amphibians possessing sentience through the cognition of stimuli without association or interpretation [333,334,335], a standard that can even extend to invertebrates [336].

In practice, the ethical use of RBCs depends on a balance between (1) the sentience of the target individuals [332,337], (2) the species’ role as a biospheric entity, thereby benefiting other moral subjects [338]; (3) support for intergenerational justice toward the environment [44,45,46]; and (4) the principle of the greatest good for the greatest number as applied to the species perpetuation [339]. Ethical considerations depend on the justification for conducting the technique, its efficiency, general applicability, and associated stress to the targeted individuals (Table 4, ibid). Once an RBC protocol or program using animals is justified, the three basic animal-ethics principles of refinement, reduction, and replacement in research or application can be applied [340,341,342]. More research on stress during confinement and handling is needed to inform ethical standards of amphibian RBCs [163].

Refinement can be achieved by preferentially using environmental simulation to promote mating and spawning in CBPs [211,331], and through procedures that minimize stress and pain during the collection of sperm and oocytes (Section 3.2, [254,343]). Reduction can be achieved through reducing the number of individuals in CBPs by using stored sperm and by avoiding extensive hormonal stimulation trials to optimize hormonal sperm collection when large yields of sperm could be reliably collected from testicular tissues (Section 3.2). Replacement can be achieved by programs maintaining genetic diversity through biobanking of germplasms, somatic cells, or tissues for use in CBPs and in supplementation, repopulation, or assisted gene-flow programs (Section 3.2, [3,75,76]), and through optimization of these activities through genetic and demographic modeling (Section 2.2).

Biotechnologies for sperm collection using the relatively stressful technique of hormonal stimulation, when compared to collection from testicular tissue, have been subject to extensive research involving more than 40 species [61,343]. However, hormonal stimulation requires the handling of individuals, and cannulation over extended periods generally only yields low sperm numbers and concentrations [235]. Furthermore, dose-response curves for two hormones are recommended to optimize hormonal stimulation before its general use for a species [10], which requires at least 32 experimental individuals. However, if mature males are available, high sperm yields can be collected through the reliable low-stress technique of testicular tissue to provide the sperm numbers for the fertilization of large numbers of oocytes (Section 3.2). In either case, even a few biobanked sperm can provide genotypic variation or produce mature adults to provide large numbers of progeny.

Despite these ethical limitations, the collection of spermous urine through cannulation is critical and justified to provide sperm known to be equivalent to those expressed in natural spawning for the basic science of spermatology and its contribution to amphibian RBCs [266]. Spermatology is dependent on knowledge of sperm structure, physiology, and motility mechanisms, particularly with respect to viscosities found in internally fertilizing amphibians and the physical structure of oocyte gel and female sperm-choice mechanisms [71]. Institutional animal-ethics requirements should be sympathetic and flexible toward the critical need for further research to develop amphibian spermatology and for the development of RBCs to perpetuate amphibian biodiversity.

Besides animal welfare, the ethics of amphibian RBCs extend to human/animal interactions through emotional and social engagement and the broad ecological impacts of repopulations or translocations [3,81,82,334,335,344]. Furthermore, speciesism, where a preference for biobanking a species is based on political, institutional, or charismatic criteria rather than on conservation or phylogenetic status requires ethical scrutiny [206]. There is considerable engagement of the theological community in the need to support biodiversity conservation [345], and avoiding language that may be theologically misinterpreted, such as “resurrection” [85] for species restoration and “playing God”, helps to ensure broader public support [85,346,347,348]. Theologies can also contribute to biospheric sustainability through social and ethical models that respect nature [349]. Although subject to ethical scrutiny with respect to speciesism, the popularity of species restoration is shown by the global reach of the Colossus de-extinction project [307], and ethical considerations include its potential for biospheric sustainability, profound community outreach, and its financial and scientific support of the field conservation of threatened species [3,344].

## 6. Current and Future Applications

The development of amphibian RBCs has progressed to the collection and storage of anuran and salamander sperm and oocytes and their use for in vitro fertilization to produce sexually mature adults [71,86,87,233]. These RBCs can support either institutional or private CBPs, along with repopulation or translocation programs [3] and assisted gene flow (Section 2.2) However, costly programs for wild populations should be highly targeted toward ecologically, phylogenetically, or culturally significant species that are likely to eventually independently survive in the wild for at least decades [61,126,206]. This targeting is particularly relevant in consideration of the rapid and accelerating anthropogenic modification of the biosphere (See Section 1).

Species not expected to survive in the wild, irrespective of the environmental targets of COP 15, 16 [2,3], or COP 28 [3] should be subject to CBPs supported by biobanked sperm or perpetuated solely in biobanks (see Section 3). Only through addressing these issues will the amphibian RBC community satisfy their ethical responsibilities toward providing reliable and cost-effective intergenerational justice. Reproduction has been well studied in only about 5% of caecilian species, and we must first learn more about their basic process of reproduction and its controls before we can generalize artificial augmentations [350]. Therefore, caecilian species offer exceptional challenges and opportunities for the development and application of RBCs because of many species’ internal fertilization and development and the maternal care of juveniles in all terrestrial species [71,89,90,91].

The high costs of implementing recovery-based conservation are attributed to maintaining or providing specialized habitats, extensive research and monitoring, conservation breeding and reintroduction programs, and the need for professional management [3,351]. The financial burden of meeting these global biodiversity conservation targets by reducing extinction risk through interventions along with establishing and maintaining protected areas is substantial, with estimates of billions a year [352], with mammals and birds typically requiring the most resources [353]. To address these challenges, a globally inclusive approach is needed, focusing on developing highly cost-efficient RBC facilities in regions with high amphibian diversity that welcome community engagement and international collaborations [3,354].

The short-term prevention of the extinction of amphibian species through RBCs to maintain, repopulate, or translocate amphibian populations in the wild [3] has already been reliably and economically demonstrated. Between 2007 and 2017, there was a ~60% increase in amphibian CBPs, including 80 species, or 2.9% of all species, with half of these CBPs involving repopulation or augmentation and 70% having some success [10,61]. However, these approaches must recognize a future of dramatic ecosystem collapses driven by global heating leading to mass extinctions [13,47,48,49,54]. The establishment of biobanks of amphibian cells and tissues will provide cost-effective and reliable species perpetuation [3,68,77,78,79,80,81,82,83].

Exciting opportunities for amphibian RBC research and development include cloning and somatic-cell techniques for species restoration (de-extinction) [323]), extraterrestrial biotechnologies [3,355,356], and terraforming for colonization [3,355]. Terraforming conceptually includes the past, current, and future anthropogenic modification of Earth’s biosphere as extended to potentially interplanetary and interstellar extraterrestrial ecosystems along with their biodiversity [3,355].

Public outreach for amphibian conservation includes educational programs, community engagement, media campaigns, and interactive experiences [357] to foster empathy, promote citizen science, and encourage sustainable practices [3,61,64,358]. Perceptions of conservation success are generally perceived in terms of species and habitat improvements, effective program management, and the application of science-based conservation including RBCs [61,358].

The 2024 ACAP provides a guide for capturing outcomes, identifying gaps, and measuring progress in conservation efforts [61]. However, the ACAP framework was limited to supporting “*Amphibians thriving in nature*” (in the wild), along with the Amphibian Ark neglecting species that cannot foreseeably be returned to the wild [359], and both generally disregard transformational changes to species management [3]. “Chapter 12, *Amphibian assisted reproductive technologies and biobanking*” of the 2024 ACAP, in the “*Priorities and recommendation”* section, does not mention species perpetuation through biobanking of somatic cells or tissues, cloning, and species restoration [61]. Furthermore, the 2024 ACAP “*Chapter 11. Conservation breeding*” of the 2024 ACAP is not inclusive of private caregivers’ potential to contribute to the perpetuation of amphibian biodiversity [64]. The disregard of many internet sources for the benefits of the biotechnical aspects of amphibian RBCs and biobanking [360] shows the challenges facing the effective advocation and popularization of the full potential of RBCs.

The advocation and popularization of amphibian RBCs include engaging cultural discourses involving assisted evolution [361], cloning and species restoration [82,362], synthetic biology [355,363,364], and theological debates about humanity’s relationship to nature through synthetic biology, “resurrecting” species, “playing God” [361,362,363,364], and utilizing scientifically based terminologies popularized through the public media [3,61,105,106,107,271,365]. Solid RBC career streams will attract ambitious and talented researchers and activists to build RBC projects by successfully competing for influence and resources [3,105]. Initiatives should focus on species perpetuation to ameliorate amphibian mass extinction and be inclusive of the broadest global community [3,345]. Community popularization of advanced reproduction technologies is exemplified by the highly successful Colossal project, where their central theme of de-extinction (species restoration) is extended for their target species to community-based field-conservation projects [323,344], advocating bioengineering as an initiative to help heal the Earth [366,367].

## 7. Conclusions

We have shown that the perpetuation of amphibian biodiversity, rather than focusing on the unachievable goals of many amphibians thriving in the wild, should recognize the community’s responsibility with respect to intergenerational justice to adapt to the environmental and cultural realities of the Anthropocene [368,369], including the sixth mass extinction [6,7,15,47,368] and humanity’s interplanetary and interstellar colonization. This transformation should harness the potential of biobanking of germplasm, somatic cells, or tissues and species restoration to perpetuate species and be inclusive of all global participants [3,64]. A greater focus is also needed to garner global support and international engagement toward developing RBCs in highly biodiverse regions, especially developing countries, and the provision of independent finance. An independent, democratic, and inclusive global organization representing an increasingly assertive multipolar world is needed to advocate these needs [3]. Finally, will humanity, over the coming millennia, look back to this century relegating plausibly thousands of amphibian species that will not continue to exist in the wild to extinction [359,370]? Or, alternatively, will we face the challenges of the Anthropocene presented by inevitable biospheric modification, especially through global heating [48,49], and perpetuate species through realist innovative, cost-effective, and reliable biotechnologies [3] supported by broader community inclusion in a multipolar world [3,53]?

## Figures and Tables

**Table 1:** Alternative approaches to the numbers of biobanked founder individuals for maintaining ex situ genetic diversity. M = male, F = female, CBP = conservation breeding program.

Population	M	F	Application	Year	Reference
Metapopulation	5	5	27 threatened species.	2005	[133]
Metapopulation	28	25	CBP	2007	[120]
Metapopulation	25	-	CBP	2012	[134]
Fragmented/sub-pop.	10	-	AGF 4 years, depending on kinship	2022	[135]
Geographic clusters	10	20	Geographic clusters	2022	[130]
Metapopulation	20+	20+	CBP	2024	[118]

**Table 2:** The number of individuals to be housed in a CBP to maintain 90% of the original genetic diversity from 25 male and 25 female founders over programs of 25 years, based on age to maturity, longevity, and individual studbook management (Individual) or group management (Group). * = Minimum population sizes of more than 100 are recommended to allow for unexpected loss of individuals [120]. # = Only group management because of short longevity.

Examples of Genera	Age to Maturity	Longevity	Individual	Group
*Acris*	<1 year	1 year	1590	#
*Eleutherodactylus*, *Nectophrynoides*, some *Hyperoliidae*	<1 year	2–5 years	400	400
*Hylidae*, some *Hyperoliidae*, *Scaphiophryne*	1–5 years	<5 years	135	265
*Dendrobatidae*, *Typhlonectes*, *Tylototriton/Echinotriton*, *Theloderma*, *Cynops*, *Leptodactylus*, *Ceratobatrachus*, *Mantella*, *Atelopus*	1–5 years	5–15 years	70 *	140
*Salamandra*, some *Ambystoma*	1–5 years	>15 years	60 *	80 *
*Cryptobranchus*, *Andrias*	>5 years	>15 years	45 *	80 *

**Table 3:** Heterocytoplasmic somatic cell nuclear transfer (SCNT) cloning in amphibians.

Nucleus Donors	Recipients	Results	Year	Ref
*Rana pipiens*	*Aquarana* (*Rana*) *catesbeiana*	Died late blastula	1952	[297]
*R. n. brevipoda*	*R.n. nigromaculata*	Metamorphs	1957	[298]
*R. pipiens*	*R. sylvatica*	Late blastula/early neurula	1958	[299]
*R. n. nigromaculata*	*R. n. brevipoda*	Adults	1961	[300]
*R. pipiens*	*R. palustris*	Post-neurula	1963	[301]
*R. n nigromaculata*	*R. n. brevipoda*	Adults–F1 reproduction	1963	[97,98]
*R. japonica*	*R. ornativentris*	F2	1963	[302]
*R. nigromaculata* *R. temporaria*	*R. brevipoda* *R. japonica*	F3	1972	[303]
*R. japonica* *R. temporaria*	*R. temporaria* *R. japonica*	Adults	1972	[304]
*R. brevipoda* *R. plancyi* *R. brevipoda* *R. esculenta*	*R. plancyi* *R. brevipoda* *R. esculenta* *R. brevipoda*	Adults	1972	[305]
*Pleurodeles waltlii* *P. poireti*	*P. poireti* *P. waltlii*	Adults	1971	[306]
*P. waltlii* *P. poireti*	*P. poireti* *P. waltlii*	Adults	1972	[307]

**Table 4:** Ethical considerations with techniques for natural or induced mating and spawning and the collection of sperm or oocytes. Note: there has been little research on stress through the handling of amphibians. Consequently, our estimates of stress are simply relative to the alternative techniques. However, even confinement in boxes alone, without regrading other handling, as used for hormonal stimulation, causes measurable stress [163].

Technique	Injection	Application Stress	Collection Technique	Collection Stress	Yield	Donor Size Limitation	References
Mating and spawning							
Natural mating and spawning	None	None	Fertilized eggs	None	Very high	None	[214,215]
Hormonally induced mating and spawning	Yes	Moderate	Fertilized eggs	None	Very high	None	[74,224]
Oocyte collection							
Hormonal stimulation oocytes with spawning into solutions	Yes	Moderate	Spawning into physiological saline	Low	High to very high	None	[243,257]
Hormonally stimulated ovarian oocytes	Yes	Moderate	Abdominal massage or cannulation	High	Low	Avoid for small species	[74,243,247,255,256,257]
Excision of hormonally stimulated ovarian oocytes	Yes	Moderate	Direct sampling	None	Very high	None	[243,257].
Sperm collection							
Collection from testicular tissue	Yes	Moderate	Macerating testicular tissue	None	Very High	None	[71,74,76,86,95,96,231,232,233]
Hormonal stimulation sperm	Yes	Moderate	Urination	Moderate	High to none	Avoid for small species	[243,257]
Hormonal stimulation sperm	Yes	Moderate	Cannulation	High	Moderate—very low	Avoid for small species	[61,75]
Hormonal stimulation sperm—topical, nasal, oral, or food item	None	Low	Cannulation	High	Moderate—very low	Possibly for small species	[71,251,252]

## Data Availability

Data concerning oocyte numbers and sizes are currently being used to support several research articles on the evolutionary ecology of amphibian sperm. Nevertheless, the data presented in this study are available, in confidence, on request to the corresponding author.

## References

[1] Lee H., Romero J., IPCC, Core Writing Team (2023). Summary for Policymakers. Climate Change 2023: Synthesis Report. Contribution of Working Groups I, II and III to the Sixth Assessment Report of the Intergovernmental Panel on Climate Change.

[2] UNEP (2024). UN Biodiversity Conference (COP 16). October 21st to 1st November. https://www.cbd.int/conferences/2024.

[3] Browne R.K., Luo Q., Wang P., Mansour N., Kaurova S.A., Gakhova E.N., Shishova N.V., Uteshev V.K., Kramarova L.I., Venu G. (2024). Ecological Civilization COP 15, COP 28, and Amphibian Sustainability through Reproduction Biotechnologies, Biobanking, and Conservation Breeding Programs (RBCs). Animals.

[4] Conradi T., Eggli U., Kreft H., Schweiger A.H., Weigelt P., Higgins S.I. (2024). Reassessment of the Risks of Climate Change for Terrestrial Ecosystems. Nat. Ecol. Evol..

[5] Grant E.H., Amburgey S.M., Gratwicke B., Chaves V.A., Belasen A.M., Bickford D., Bruhl C.A., Calatayud N.E., Clemann N., Clulow S. (2023). Amphibian Conservation in the Anthropocene. Biol. Conserv..

[6] Willcock S., Cooper G.S., Addy J., Dearing J.A. (2023). Earlier Collapse of Anthropocene Ecosystems Driven by Multiple Faster and Noisier Drivers. Nat. Sustain..

[7] Finn C., Gratarolla F., Pincheira-Donoso D. (2023). More Losers than Winners: Investigating Anthropocene Defaunation through the Diversity of Population Trends. Biol. Rev..

[8] Bradshaw C.J.A., Ehrlich P.R., Beattie A., Ceballos G., Crist E., Diamond J., Dirzo R., Ehrlich A.H., Harte J., Harte M.E. (2021). Underestimating the Challenges of Avoiding a Ghastly Future. Front. Conserv. Sci..

[9] The IUCN Red List of Threatened Species. Version 2022-2. https://www.iucnredlist.org.

[10] Silla A.J., Byrne P.G. (2019). The Role of Reproductive Technologies in Amphibian Conservation Breeding Programs. Annu. Rev. Anim. Biosci..

[11] AmphibiaWeb. https://amphibiaweb.org.

[12] Smith D., Abeli T., Bruns E.B., Dalrymple S.E., Foster J., Gilbert T.C., Hogg C.J., Lloyd N.A., Meyer A., Moehrenschlager A. (2023). Extinct in the wild: The precarious state of Earth’s most threatened group of species. Science.

[13] Proceedings of the UNFCCC COP 28. UN Climate Change Conference.

[14] Hansen J.E., Sato M., Simons L., Nazarenko L.S., Sangha I., Kharecha P., Zachos J.C., von Schuckmann K., Loeb N.G., Osman M.B. (2023). Global Warming in the Pipeline. Oxf. Open Clim. Chang..

[15] Global Tipping Points. https://global-tipping-points.org/.

[16] CAT Climate Action Tracker IMF. https://climateactiontracker.org/.

[17] Smith S.M., Geden O., Gidden M.J., Lamb W.F., Nemet G.F., Minx J.C., Buck H., Burke J., Cox E., Browne M.R. (2024). The State of Carbon Dioxide Removal. 2nd Edition. TI—The State of Carbon Dioxide Removal. https://osf.io/f85qj/https://www.stateofcdr.org/.

[18] GHR (2023). Global Hydrogen Review.

[19] Juárez-Orozco S.M., Siebe C., Fernández y Fernández D. (2017). Causes and Effects of Forest Fires in Tropical Rainforests: A Bibliometric Approach. Trop. Conserv. Sci..

[20] Zheng B., Ciais P., Chevallier F., Yang H., Canadell J.G., Chen Y., van der Velde I.R., Aben I., Chuvieco E., Davis S.J. (2023). Record-high CO_2_ emissions from boreal fires in 2021. Science.

[21] Hong X., Liu C., Zhang C., Tian Y., Wu H., Yin H., Zhu Y., Yafang Cheng Y. (2023). Vast ecosystem disturbance in a warming climate may jeopardize our climate goal of reducing CO2: A case study for megafires in the Australian ‘black summer’. Sci. Total Environ..

[22] Chikamoto M.O., DiNezio P., Lovenduski N. (2023). Long-Term Slowdown of Ocean Carbon Uptake by Alkalinity Dynamics. Geophys. Res. Lett..

[23] World Ocean Review The Role of the Ocean in the Global Carbon Cycle. https://worldoceanreview.com/en/wor-8/the-role-of-the-ocean-in-the-global-carbon-cyclee/how-the-ocean-absorbs-carbon-dioxide/.

[24] Smolders E.J.V., Western R.M., Dijkstra H.A. Probability Estimates of a 21st Century AMOC Collapse. https://arxiv.org/html/2406.11738v1.

[25] Luedtke J.A., Chanson J., Neam K., Hobin L., Maciel A.O., Catenazzi A., Borzée A., Hamidy A., Aowphol A., Jean A. (2023). Ongoing Declines for the World’s Amphibians in the Face of Emerging Threats. Nature.

[26] Guirguis J., Goodyear L.E.B., Finn C., Johnson J.V., Pincheira-Donoso D. (2023). Risk of Extinction Increases towards Higher Elevations Across the World’s Amphibians. Glob. Ecol. Biogeogr..

[27] Zhang W., Huang D., Wang R., Liu J., Du N. (2016). Altitudinal Patterns of Species Diversity and Phylogenetic Diversity across Temperate Mountain Forests of Northern China. PLoS ONE.

[28] Khatiwada J.R., Zhao T., Chen Y., Wang B., Xei F., Cannatella D.C., Jiang J. (2019). Amphibian Community Structure along Elevation Gradients in Eastern Nepal Himalaya. BMC Ecol..

[29] Rastegar-Pouyani N., Gholamifard A., Karamiani R., Bahmani Z., Mobaraki A., Abtin E., Faizi H., Heidari N., Takesh M., Sayyadi F. (2015). Browne RK. Sustainable Management of the Herpetofauna of the Iranian Plateau and coastal Iran. Amphib. Rept. Conser..

[30] Moreira L.F., Smaniotto N.P., Ceron K., Santana D.J., Ferreira V.L., Strüssmann C., Galatti U. (2023). Ashes Still Smoking: The Influence of Fire and Land Cover on Pantanal Ecoregion Amphibians. Amphibia-Reptilia.

[31] Tomas W.M., Berlinck C.N., Chiaravalloti R.M., Faggioni G.P., Strüssmann C., Libonati R., Abrahão C.R., do Valle Alvarenga G., de Faria Bacellar A.E., de Queiroz Batista F.R. (2021). Distance Sampling Surveys Reveal 17 Million Vertebrates Directly Killed by the 2020’s Wildfires in the Pantanal, Brazil. Sci. Rep..

[32] Anjos A.G., Alvarado S.T., Solé M., Benchimol M. (2024). Influence of Fire Regime on the Taxonomic and Phylogenetic Diversity of Frog Communities in a Fire-prone Brazilian Ecosystem. For. Ecol. Manag..

[33] Trew B.T., Edwards D.P., Klinges D.H., Early R., Svátel M., Plichta R., Mayula R., Okello J., Niessner A., Barthel M. (2024). Novel Temperatures are Already Widespread Beneath the World’ Tropical Rainforests. Nat. Clim. Chang..

[34] Wu N.C., Bovo R.P., Enriquez-Urzelai U., Clusella-Trullas S., Kearney M.R., Navas C.A., Kong J.D. (2024). Global Exposure Risk of Frogs to Increasing Environmental Dryness. Nat. Clim. Chang..

[35] Godfray H.C.J., Stephens A.E.A., Jepson P.D., Jobling S., Johnson A.C., Matthiessen P., Sumpter J.P., Tyler C.R., McLean A.R. (2019). A Restatement of the Natural Science Evidence Base on the Effects of Endocrine Disrupting Chemicals on Wildlife. Proc. R. Soc. B.

[36] Sun N., Shi H., Li X., Gao C., Liu R. (2023). Combined Toxicity of Micro/nanoplastics Loaded with Environmental Pollutants to Organisms and Cells: Role, Effects, and Mechanism. Environ. Int..

[37] Christensen K. (2024). Thawing Permafrost Releases Industrial Contaminants into Arctic Communities. Environ Health Perspect..

[38] Weinhold B. (2009). Persistent Organic Pollutants: Melting Glaciers Release Frozen Toxicants. Environ. Health Perspect..

[39] McComb B.C., Cushman S.A. (2020). Synergistic Effects of Pervasive Stressors on Ecosystems and Biodiversity. Front. Ecol. Evol..

[40] Blake J.G., Loiselle B.A. (2024). Sharp Declines in Observation and Capture Rates of Amazon Birds in Absence of Human Disturbance. Glob. Ecol. Conserv..

[41] Campbell L.G., Anderson K.A., Marcec-Greaves R., Cardoso P., Barton P.S., Birkhofer K., Chichorro F., Deacon C., Fartmann T., Fukushima C.S. (2020). Scientists’ Warning to Humanity on Insect Extinctions. Biol. Conserv..

[42] Precautionary Principle International Institute for Sustainable Development. https://www.iisd.org/articles/deep-dive/precautionary-principle.

[43] Gergis J. An Intergenerational Crime Against Humanity What Will It Take for Political Leaders to Start Taking Climate Change Seriously. https://theconversation.com/an-intergenerational-crime-against-humanity-what-will-it-take-for-political-leaders-to-start-taking-climate-change-seriously-231383.

[44] Barry B. (1997). Sustainability and Intergenerational Justice. Theoria.

[45] Spanning R., Knapp G., Krall H. (2021). Youth in the Anthropocene: Questions of Intergenerational Justice and Learning in a More-Than-Human World. Youth Cultures in a Globalized World.

[46] Pereira L.M., Smith S.R., Gifford L., Newell P., Smith B., Villasante S., Achieng T., Castro A., Constantino S.M., Ghadiali A. (2023). Risks, Ethics and Justice in the governance of positive tipping points. EGUsphere.

[47] Radchuk V., Reed T., Teplitsky C., van de Pol M., Charmantiet A., Hassall C., Adamik P., Adriaensen F., Ahola M.P., Arcese P. (2019). Adaptive Responses of Animals to Climate Change are Most Likely Insufficient. Nat. Commun..

[48] Treves A., Artelle K.A., Darimont C.T., Lynn W.S., Paquet P., Santiago-Ávila F.J., Shaw R., Wood M.C. (2018). Intergenerational Equity can Help to Prevent Climate Change and Extinction. Nat. Ecol. Evol..

[49] Méjean A., Pottier A., Zuber S., Fleurbaey M. Intergenerational Equity Under Catastrophic Climate Change. Global Priorities Institute Working Paper No. 5-2020. https://globalprioritiesinstitute.org/wp-content/uploads/M%C3%A9jean-et-al_Intergenerational-equity-under-catastrophic-climate-change.pdf.

[50] Fedele G., Donatti C.I., Harvey C.A., Hannah L., Hole D.G. (2019). Transformative Adaptation to Climate Change for Sustainable Social-ecological Systems. Environ. Sci. Policy.

[51] O’Brien K. (2012). Global Environmental Change II: From Adaptation to Deliberate Transformation. Hum. Geogr. J..

[52] Bolam F.C., Ahumada J., Akçakaya H.R., Brooks T.M., Elliott W., Hoban S., Mair L., Mallon D., McGowan P.J.K., Raimondo D. (2020). Preventing Extinctions Post-2020 Requires Recovery Actions and Transformative Change. BioRxiv.

[53] Bowman M., Bowman M., Davies P., Goodwin E. (2016). Chapter 1 Law, Legal Scholarship and the Conservation of Biological Diversity: 2020 Vision and Beyond. Research Handbook on Biodiversity and Law.

[54] Senior R.A., Bagwyn R., Leng D., Killion A.K., Jetz W., Wilcove D.S. (2024). Global Shortfalls in Documented Actions to Conserve Biodiversity. Nature.

[55] Bateman I., Balmford A. (2023). Current Conservation Policies Risk Accelerating Biodiversity Loss. Nature.

[56] Palacio R.D., Abarca M., Armenteras D., Balza U., Dollar L., Froese G.Z.L., Galligan M.P., Gula J., Giordano A.J., Jacobson A.P. (2023). The Global Influence of the IUCN Red List can Hinder Species Conservation Efforts. Authorea Prepr..

[57] Crutzen P.J., Stoemer E.F. (2000). The “Anthropocene”. The International Geosphere–Biosphere Programme (IGBP): A Study of Global Change of the International Council for Science (ICSU) Newsletter. Glob. Chang. Newsl..

[58] United Nations Convention on Biodiversity. https://www.un.org/en/observances/biological-diversity-day/convention.

[59] Byers O., Lees C., Wilcken J., Schwitzer C. (2013). The One Plan Approach: The Philosophy and Implementation of CBSG’s Approach to Integrated Species Conservation Planning. WAZA Mag..

[60] Ziegler T. The IUCN/SSC CPSG’s One Plan Approach and the Role of Progressive Zoos in Conservation: Case Studies from Herpetology. Proceedings of the 14th National Congress of the Italian Society for Herpetology.

[61] Wren S., Borzée A., Marcec-Greaves R., Angulo A., IUCN SSC Amphibian Specialist Group (2024). Amphibian Conservation Action Plan: A Status Review and Roadmap for Global Amphibian Conservation.

[62] AArk Amphibian Ark. www.amphibianark.org/.

[63] Zippel K., Johnson K., Agliardo R.G., Gibson R., McFadden M., Browne R., Martinez C.M., Townsend E. (2011). The Amphibian ARK: A Global Community for Ex situ Conservation of Amphibians. Herpetol. Conserv. Biol..

[64] Browne R.K., Janzen P., Bagaturov M.F., van Houte D.K. (2018). Amphibian Keeper Conservation Breeding Programs. J. Zool. Res..

[65] Responsible Herpetological Project Conservation Breeding Programs. https://responsibleherpetoculture.foundation/.

[66] Pizzutto C.S., Colbachini H., Jorge-Neto P.N. (2021). One Conservation: The Integrated View of Biodiversity Conservation. Anim. Reprod..

[67] Thomas-Walters L., McCallum J., Montgomery R., Petros C., Wan A.K.Y., Veríssimo D. (2023). Systematic review of conservation interventions to promote voluntary behavior change. Conserv. Biol..

[68] Hagedorn M., Parenti L.R., Craddock R.A., Comizzoli P., Mabee P., Meinke B., Wolf S.M., Bischof J.C., Sandlin R.D., Tessier S.N. (2024). Safeguarding Earth’s biodiversity by creating a lunar biorepository. BioScience.

[69] Della-Tonga G., Howell L.G., Clulow J., Langhorne C.J., Marcec-Greaves R., Calatayud N.E. (2020). Evaluating Amphibian Biobanking and Reproduction for Captive Breeding Programs According to the Amphibian Conservation Action Plan Objectives. Theriogenology.

[70] Clulow J., Mahony M., Browne R., Pomering M., Clark A., Campbell A. (1999). Applications of Assisted Reproductive Technologies (ART) to Endangered Anuran Amphibians. Declines and Disappearances of Australian Frogs.

[71] Browne R.K., Kaurova S.A., Vasudevan K., McGinnity D., Venu G., Gonzalez M., Uteshev V.K., Marcec-Greaves R. (2022). Reproduction Technologies for the Sustainable Management of Caudata (salamander) and Gymnophiona (Caecilian) biodiversity. Reprod. Fertil. Dev..

[72] Ananjeva N.B., Uteshev V.K., Orlov N.L., Ryabov S.A., Gakhova E.N., Kaurova S.A., Kramarova L.I., Shishova N.V., Browne R.K. (2017). Comparison of the Modern Reproductive Technologies for Amphibians and Reptiles. Russ. J. Herpetol..

[73] Clulow J., Upton R., Trudeau V.L., Clulow S. (2019). Amphibian Assisted Reproductive Technologies: Moving from Technology to Application. Adv. Exp. Med. Biol..

[74] Uteshev V.K., Gakhova E.N., Kramarova L.I., Shishova N.V., Kaurova S.A., Kidova E.A., Kidov A.A., Browne R.K. (2023). Russian Collaborative Development of Reproduction Technologies for the Sustainable Management of Amphibian Biodiversity. Asian Herpetol. Res..

[75] Anastas Z.M., Byrne P.G., O’Brien J.K., Hobbs R.J., Upton R., Silla A.J. (2023). The Increasing Role of Short-Term Sperm Storage and Cryopreservation in Conserving Threatened Amphibian Species. Animals.

[76] Browne R.K., Silla A.J., Upton R., Della-Togna G., Marcec-Greaves R., Shishova N.V., Uteshev V.K., Proano B., Pe-rez O.D., Mansour N. (2019). Sperm Collection and Storage for the Sustainable Management of Amphibian Biodiversity. Theriogenology.

[77] Bolton R.L., Mooney A., Pettit M.T., Bolton A.E., Morgan L., Drake G.J., Appeltant R., Walker S.L., Gillis J.D., Hvilsom C. (2022). Resurrecting Biodiversity: Advanced Assisted Reproductive Technologies and Biobanking. Reprod. Fertil..

[78] Seddon P.J., Griffiths C.J., Soorae P.S., Armstrong D.P. (2014). Reversing Defaunation: Restoring Species in a Changing World. Science.

[79] Strand J., Thomsen H., Jensen J.B., Marcussen C., Nicolajsen T.B., Skriver M.B., Søgaard I.M., Ezaz T., Purup S., Callesen H. (2020). Biobanking in Amphibian and Reptilian Conservation and Management: Opportunities and Challenges. Conserv. Gen. Res..

[80] Strand J., Fraser B., Houck M.L., Clulow S., Silla A.J., Kouba A.J., Heatwole H. (2022). Culturing and Biobanking of Amphibian Cell Lines for Conservation Applications. Reproductive Technologies and Biobanking for the Conservation of Amphibians.

[81] Mooney A., Ryder O.A., Houck M.L., Staerk J., Conde D.A., Buckley Y.M. (2023). Maximizing the Potential for Living Cell Banks to Contribute to Global Conservation Priorities. Zoo Biol..

[82] Cowl V.B., Comizzoli P., Appeltant R., Bolton R.L., Browne R.K., Holt W.V., Penfold L.M., Swegen A., Walker S.L., Williams S.A. (2024). Cloning for the Twenty-First Century and Its Place in Endangered Species Conservation. Annu. Rev. Anim. Biosci..

[83] Lermen D., Blömeke B., Browne R.K., Clarke A., Dyce P.W., Fixemer T., Fuhr G.R., Holt W.V., Jewgenow K., Lloyd R.E. (2009). Meeting Review. Cryobanking of Viable Biomaterials: Implementation of New Strategies for Conservation Purposes. Mol. Ecol..

[84] Bowgen K.M., Kettel E.F., Butchart S.H.M., Carr J.A., Foden W.B., Magin G., Morecroft M.D., Smith R.K., Stein B.A., Sutherland W.J. (2022). Conservation interventions can benefit species impacted by climate change. Biol. Conserv..

[85] Mastromonaco G.F., Songsasen N., Giorgio A., Presice G.A. (2020). Chapter 7—Reproductive Technologies for the Conservation of Wildlife and Endangered Species, In Reproductive Technologies in Animals.

[86] Upton R., Clulow S., Calatayud N.E., Colyvas K., Seeto R.G.Y., Wong L.A.M., Mahony M.J., Clulow J. (2021). Generation of Reproductively Mature Offspring from the Endangered Green and Golden Bell Frog *Litoria aurea* using Cryopreserved Spermatozoa. Reprod. Fertil. Dev..

[87] Lampert S.S., Burger I.J., Julien A.R., Gillis A.B., Kouba A.J., Barber D., Kouba C.K. (2023). Sperm Cryopreservation as a Tool for Amphibian Conservation: Production of F2 Generation Offspring from Cryo-Produced F1 Progeny. Animals.

[88] Holmes B., Ziermann J.M., Strzelecki A., Springer S., Zieger M. (2024). Who notices Gymnophiona? Google Trends data reveal interesting trends for recent amphibian species. Ecol. Complex..

[89] Venu G., Raju N.G., Wilkinson M., Browne R.K., Varadh K., Balakrishna G.N., Ramakrishna S., Venkatachalaiah G. (2020). First records of the Long-headed Caecilian, *Ichthyophis longicephalus* Pillai, 1986 (Gymnophiona: Ichthyophiidae) from the states of Karnataka and Tamil Nadu, India with comments on its conservation status. JAD.

[90] Reinhard S., Kupfer A. (2022). Maternal investment in the viviparous caecilian amphibian *Typhlonectes natans* (Gymnophiona: Typhlonectidae). Zool. Anz..

[91] Kuehnel S., Kupfer A. (2012). Sperm Storage in Caecilian Amphibians. Front. Zool..

[92] Parmley D. General Husbandry of Terrestrial Caecilians. https://www.caudata.org/cc/articles/caecilian_care_Parmley.pdf.

[93] Kaurova S.A., Chekurova N.R., Melnikova E.V., Uteshev V.K., Gakhova E.N., Gakhova E.N., Karnaukhov V.N. (1996). Cryopreservation of Frog *Rana temporaria* Sperm without Loss of Fertilizing Capacity. Proceedings of the 14th Working Meeting.

[94] Kaurova S.A., Uteshev V.K., Chekurova N.R., Gakhova E.N. (1997). Cryopreservation of Testis of Frog *Rana temporaria*. Infusionsther Transfusionsmed.

[95] Browne R.K., Clulow J., Mahony M., Clark A. (1998). Successful Recovery of Motility and Fertility of Cryopreserved Cane Toad (*Bufo marinus*) sperm. Cryobiology.

[96] Browne R.K., Clulow J., Mahony M. (2001). Short-term Storage of Cane Toad (*Bufo marinus*) Gametes. Reproduction.

[97] Karamura T., Nishioka M. (1963). Reciprocal Diploid Nucleocytoplasmic Hybrids between Two Species of Japanese Pond Frogs and their Offspring. J. Sci. Hiroshima Univ..

[98] Karamura T., Nishioka M. (1963). Nucleo-cytoplasmic Hybrid Frog between Two Species of Japanese Brown Frogs and their Offspring. J. Sci. Hiroshima Univ..

[99] Kouba C.K., Julien A.R., Silla A.J., Kouba A.J., Heatwole H. (2022). Linking In situ and Ex situ Populations of Threatened Amphibian Species using Genome Resource Banks. Reproductive Technologies and Biobanking for the Conservation of Amphibians.

[100] (2023). Taronga Conservation Society Australia. https://taronga.org.au/conservation-and-science/current-research/frog-conservation-biobanking.

[101] Panama Amphibian Conservation and Rescue Project. http://amphibianrescue.org/.

[102] Burger I., Julien A.R., Kouba A.J., Councell K.R., Barber B., Pacheco C., Kouba C.K. (2021). Linking *In Situ* and *Ex Situ* Populations of the Endangered Puerto Rican Crested Toad. Conserv. Sci. Pract..

[103] Wyoming Toad Sees Recovery in the Southwest Wyoming Game and Fish Department. https://wgfd.wyo.gov/News/Wyoming-toad-recovery-sees-success-in-southeast.

[104] Northern Corroboree Frog Recovery Program Australia’s Nature Hub. https://www.australiasnaturehub.gov.au/action-inventory/northern-corroboree-frog-recovery-program.

[105] Browne R.K., Silla A.J., Kouba A.J., Heatwole H. (2022). Reproductive Technologies and Biobanking for the Conservation of Amphibians.

[106] Martinez A., Mammola S.S. (2021). Specialized Terminology Reduces the Number of Citations of Scientific Papers. Proc. R. Soc. B Biol. Sci..

[107] Textor M., Rami D. (2015). Proper Names: Philosophical and Linguistic Perspectives. Erkenn.

[108] Chala D., Endresen D., Demissew S., Slaughter L.A., Johnsen E.B., Stenseth N.C. (2024). Address Social Injustices in Taxonomy: Implement Extended Revisions of Names with Ethical Issues and Persistent Identifiers for Tracing Name Changes. Preprints.

[109] Mc Cartney A.M., Head M.A., Tsosie K.S., Sterner B., Glass J.R., Paez S., Geary J., Hudson M. (2023). Indigenous Peoples and Local Communities as Partners in the Sequencing of Global Eukaryotic Biodiversity. NPJ Biodivers..

[110] ABS (2014). Nagoya Protocol on Access and Benefit-Sharing. https://www.cbd.int/abs/.

[111] Khoday K. (2022). Decolonising the Environment: Third World Approaches to the Planetary Crisis. Indones. J. Int. Law.

[112] Abiddin N.Z., Ibrahim I., Abdul Aziz S.A. (2022). Non-Governmental Organisations (NGOs) and their Part towards Sustainable Community Development. Sustainability.

[113] Adams W., Mulligan M. (2002). Decolonising Nature; Strategies for Conservation in a Post-Colonial Era.

[114] Frankham R. (2005). Genetics and Extinction. Biol. Conserv..

[115] Williams R.N., Bos D.H., Gopurenko D., Dewoody J.A. (2008). Amphibian Malformations and Inbreeding. Biol. Lett..

[116] Brennan R.S., Garrett A.D., Huber K.E., Hargarten H., Pespeni M.H. (2019). Rare Genetic Variation and Balanced Polymorphisms are Important for Survival in Global Change Conditions. Proc. R. Soc. B..

[117] Ralls K., Ballou J.D., Dudash M.R., Eldridge M.D.B., Fenster C.B., Lacy R.C., Sunnucks P., Frankham R. (2018). Call for a Paradigm Shift in the Genetic Management of Fragmented Populations. Conserv. Lett..

[118] AArk Founder Numbers. https://www.amphibianark.org/conservation-programs/captive-programs/founder-animals/.

[119] Johnston L.A., Lacy R.C. (1995). Genome Resource Banking for Species Conservation: Selection of Sperm Donors. Cryobiology.

[120] Schad K. (2008). Amphibian Population Management Guidelines. Proceedings of the Amphibian Population Management Workshop.

[121] Beebee T.J.C. (2005). Conservation Genetics of Amphibians. Heredity.

[122] Johnson W.E., Koepfli K., Holt W., Brown J., Comizzoli P. (2014). The Role of Genomics in Conservation and Reproductive Sciences. Reproductive Sciences in Animal Conservation.

[123] Farquharson K.A., Hogg C.J., Grueber C.E. (2021). Offspring Survival Changes over Generations of Captive Breeding. Nat. Commun..

[124] Allentoft M.E., O’Brien J. (2010). Global Amphibian Declines, Loss of Genetic Diversity and Fitness: A Review. Diversity.

[125] Hinkson K.M., Poo S. (2020). Inbreeding Depression in Sperm Quality in a Critically Endangered Amphibian. Zoo Biol..

[126] Conde D.A., Colchero F., Gusset M., Pearce-Kelly P., Byers O., Flesness N., Browne R.K., Jones O.R. (2013). Zoos through the Lens of the IUCN Red List: A Global Metapopulation Approach to Support Conservation Breeding Programs. PLoS ONE.

[127] Kardos M., Armstrong E.E., Fitzpatrick S.W., Hauser S., Hedrick P.W., Miller J.M., Tallmon D.A., Funk W.C. (2021). The Crucial Role of Genome-wide Genetic Variation in Conservation. Proc. Natl. Acad. Sci USA.

[128] Kouba A.J., Silla A.J., Kouba A.J., Heatwole H. (2022). Genome Resource Banks as a Tool for Amphibian Conservation. Reproductive Technologies and Biobanking for the Conservation of Amphibians.

[129] Onley I.R., Moseby K.E., Austin J.J. (2021). Genomic Approaches for Conservation Management in Australia under Climate Change. Life.

[130] Karthikeyan V., Barkha S., Venu G., Gowri M., Lisa G., Kishor G.B. (2022). Ex-Situ Management of Amphibians in Indian Zoos.

[131] Eskew E.A., Shock B.C., LaDouceur E.E.B., Keel K., Miller M.R., Foley J.E., Todd B.D. (2018). Gene Expression Differs in Susceptible and Resistant Amphibians Exposed to *Batrachochytrium dendrobatidis*. R. Soc. Open Sci..

[132] Hantak M.M., Kuchta S.R. (2018). Predator Perception Across Space and Time: Relative Camouflage in a Colour Polymorphic Salamander. Biol. J. Linn. Soc..

[133] Mahony M.J., Clulow J., Murray K., Skerratt L.F., Marantelli G., Berger L., Hunter D. (2011). Appendix 2. Cryopreservation and Reconstitution Technologies: A Proposal to Establish A Genome Resource Bank For Threatened Australian Amphibians. Guidelines for Minimising Disease Rrisks Associated with Captive Breeding, Rraising and Restocking Pprograms for Australian Frogs.

[134] FAO (2012). Cryoconservation of Animal Genetic Resources.

[135] Harnal V.K., Wildt D.E., Bird D.M., Monfort S.L., Ballou J.D. (2002). Computer Simulations to Determine the Efficacy of Different Genome Resource Banking Strategies for Maintaining Genetic Diversity. Cryobiology.

[136] Witzenberger K.A., Hochkirch A. (2011). Ex situ Conservation Genetics: A Review of Molecular Studies on the Genetic Consequences of Captive Breeding Programs for Endangered Animal Species. Biodivers. Conserv..

[137] Crates R., Stojanovic D., Heinsohn R. (2023). The Phenotypic Costs of Captivity. Biol. Rev. Camb. Philos. Soc..

[138] Lee J., Park J., Do Y. (2023). Importance and Application of Amphibian Sperm Cryopreservation. J. Wetl. Res..

[139] Howell L.G., Frankham R., Rodger J.C., Witt R.R., Clulow S., Upton R.M.O., Clulow J. (2021). Integrating Biobanking Minimises Inbreeding and Produces Significant Cost Benefits for a Threatened Frog Captive Breeding Programs. Conserv. Lett..

[140] Howell L.G., Mawson P.R., Frankham R., Rodger J.C., Upton R.M.O., Witt R.W., Calatayud N.E., Clulow S., Clulow J. (2021). Integrating Biobanking could Produce Significant Cost Benefits and Minimise Inbreeding for Australian Amphibian Captive Breeding Program. Reprod. Fertil. Dev..

[141] Naranjo R.E., Naydenova E., Proaño-Bolaños C., Vizuete K., Debut A., Arias M.T., Coloma L.A. (2022). Development of Assisted Reproductive Technologies for the Conservation of *Atelopus* sp. (*spumarius* complex). Cryobiology.

[142] (2023). AArk Conservation Needs Assessments. https://www.conservationneeds.org/default.aspx.

[143] Rehberg-Besler N., Doucet S.M., Mennill D.J. (2016). Vocal Behavior of the Explosively Breeding Neotropical Yellow Toad, *Incilius luetkenii*. J. Herpetol..

[144] Kupfer A., Fairbairn D.J., Wolf U., Blanckenhorn W.U., Székely T. (2007). Ch 5, Sexual size dimorphism in amphibians: An overview. Sex, Size and Gender Roles: Evolutionary Studies of Sexual Size Dimorphism.

[145] Liao W.B., Zeng Y., Yang J.D. (2013). Sexual Size Dimorphism in Anurans: Roles of Mating System and Habitat Types. Front. Zool..

[146] Bell R.C., Zamudio K.R. (2012). Sexual Dichromatism in Frogs: Natural Selection, Sexual Selection and Unexpected Diversity. Proc. Biol. Sci..

[147] Christe P., Keller L., Roulin A. (2006). The Predation Cost of Being a Male: Implications for Sex-specific Rates of Ageing. Oikos.

[148] Han X., Fu J. (2013). Does Life History Shape Sexual Size Dimorphism in Anurans? A Comparative Analysis. BMC Evol. Biol..

[149] Ryan M.J., Tuttle M., Taft L.K. (1981). The Costs and Benefits of Frog Chorusing Behavior. Behav. Ecol. Sociobiol..

[150] Watt A.M., Marcec-Greaves R., Hinkson K.M., Poo S., Roberts B., Pitcher T.E. (2021). Effects of Age on Sperm Quality Metrics in Endangered Mississippi Gopher Frogs (*Lithobates sevosus*) from Captive Populations Used for Controlled Propagation and Reintroduction Efforts. Zoo Biol..

[151] Vieites D.R., Nieto-Román S., Barluenga M., Palanca A., Vences M., Meyer A. (2004). Post-mating Clutch Piracy in an Amphibian. Nature.

[152] Borowsky R., Luk A., He X., Kim R.S. (2018). Unique Sperm Haplotypes are Associated with Phenotypically Different Sperm Subpopulations in *Astyanax* Fish. BMC Biol..

[153] Scheltinga D.M., Jamieson B.G.M., Jamieson G.M. (2013). Spermatogenesis and the Mature Spermatozoon: Form, Function and Phylogenetic Implications. Reproductive Biology and Phylogeny of Anura.

[154] Firman R.C., Gasparini C., Manier M.K., Pizzari T. (2017). Postmating Female Control: 20 Years of Cryptic Female Choice. Trends Ecol. Evol..

[155] Poo S., Bogisich A., Mack M., Lynn B.K., Devan-Song A. (2022). Post-release Comparisons of Amphibian Growth reveal Challenges with Sperm Cryopreservation as a Conservation Tool. Conserv. Sci. Pract..

[156] Hughes D.P., Libersat F. (2019). Parasite Manipulation of Host Behavior. Curr. Biol..

[157] Browne R.K., Li H., Vaughan M. (2006). Sexually Mediated Shedding of *Myxobolus fallax* Spores during Spermiation of *Litoria fallax* (anura). Dis. Aquat. Org..

[158] Brannelly L.A., Webb R., Skerratt L.F., Berger L. (2016). Amphibians with Infectious Disease Increase their Reproductive Effort: Evidence for the Terminal Investment Hypothesis. Open Biol..

[159] Hartigan A., Phalen D.N., Slapeta J. (2013). Myxosporean Parasites in Australian Frogs: Importance, Implications and Future Directions. Int. J. Parasitol. Parasites Wildl..

[160] Eiras J.C. (2005). An Overview on the Myxosporean Parasites in Amphibians and Reptiles. Acta Parasitol..

[161] Hartigan A., Phalen D.N., Šlapeta J. (2010). Museum Material Reveals a Frog Parasite Emergence after the Invasion of the Cane Toad in Australia. Parasites Vectors.

[162] Kelleher S.R., Scheele B.C., Silla A.J., Keogh S., Hunter D.A., Endler J.A., Byrne P.G. (2021). Disease influences male advertisement and mating outcomes in a critically endangered amphibian. Anim. Behav..

[163] Narayan E., Silla A.J., Kouba A.J., Heatwole H. (2022). Chapter 5. Non-invasive Monitoring of Stress Physiology During Management and Breeding of Amphibians in Captivity. Reproductive Technologies and Biobanking for the Conservation of Amphibians.

[164] Grummer J.A., Booker T.R., Matthey-Doret R., Nietlisbach P., Thomaz A.T., Whit-lock M.C. (2022). The Immediate Costs and Long-term Benefits of Assisted Gene Flow in Large Populations. Conserv. Biol..

[165] Burger I.J. (2021). The ART of Amphibian Conservation: Linking In-Situ and Ex-Situ Populations of Endangered Species Through Genome Banking. Master’s Thesis.

[166] Kelly E., Phillips B.L. (2016). Targeted Gene Flow for Conservation. Conserv. Biol..

[167] Keller L.F., Waller D.M. (2002). Inbreeding Effects in Wild Populations. Trends Ecol. Evol..

[168] Brook B.W., Tonkyn D.W., O’Grady J.J., Frankham R. (2002). Contribution of Inbreeding to Extinction Risk in Threatened Species. Conserv. Ecol..

[169] Stock S.E., Klop-Toker K., Wallace S., Kelly O., Callen A., Seeto R., Mahony S.V., Hayward M.W., Mahony M.J. (2023). Uncovering Inbreeding, Small Populations, and Strong Genetic Isolation in an Australian Threatened Frog, *Litoria littlejohni*. Conserv. Genet..

[170] Reed D.H., Frankham R. (2003). Correlation between Fitness and Genetic Diversity. Conserv. Biol..

[171] Cheptou P.O., Hargreaves A.L., Bonte D., Jacquemyn H. (2017). Adaptation to Fragmentation: Evolutionary Dynamics Driven by Human Influences. Philos. Trans. R. Soc. Lond. B Biol. Sci..

[172] Westram A.M., Stankowski S., Surendranadh P., Barton N. (2022). What is Reproductive Isolation?. J. Evol. Biol..

[173] Nistelberger H.M., Roycroft E., Macdonald A.J., McArthur S., White L.C., Grady P.G.S., Pierson J., Sims C., Cowen S., Moseby K. (2023). Genetic Mixing in Conservation Translocations Increases Diversity of a Keystone Threatened Species, *Bettongia lesueur*. Mol. Ecol..

[174] Albert E.M., Ferna’ndez-Beaskoetxea S., Godoy J.A., Tobler U., Schmidt B.R., Bosch J. (2015). Genetic Management of an Amphibian Population after Chyridiomycosis Outbreak. Conserv. Genet..

[175] Frankham R., Ballou J.D., Eldridge M.D., Lacy R.C., Ralls K., Dudash M.R., Fenster C.B. (2011). Predicting the Probability of Outbreeding Depression. Conserv. Biol..

[176] Lindsay W.R., Madsen T., Wapstra E., Lillie M., Loeb L., Ujvari B., Olsson M. (2020). Long Term Effects of Outbreeding: Experimental Founding of Island Population Eliminates Malformations and Improves Hatching Success in Sand Lizards. Biol. Conserv..

[177] Frankham R., Bradshaw C.J.A., Brook B.W. (2014). Genetics in Conservation Management: Revised Recommendations for the 50/500 rules, Red List Criteria and Population Viability Analysis. Biol. Conserv..

[178] Silla A.J., Byrne P.G. (2024). The Importance of Quantifying Fitness-determining Traits Tthroughout Life to Assess the Application of Reproductive Technologies for Amphibian Species Recovery. Front. Conserv. Sci..

[179] Scheele B.C., Hunter D.A., Skerratt L.F., Brannelly L.A., Driscoll D.A. (2015). Low Impact of Chytridiomycosis on Frog Recruitment Enables Persistence in Refuges Despite High Adult Mortality. Biol. Conserv..

[180] Sgrò C.M., Lowe A.J., Hoffmann A.A. (2011). Building Evolutionary Resilience for Conserving Biodiversity under Climate Change. Evol. Appl..

[181] Carvalho C.S., Lanes E.C.M., Silva A.R., Caldeira C.F., Carvalho-Filho N., Gastauer M., Imperatriz-Fonsecaa V.L., Nascimento W., Oliveeira G., Siqueira J.O. (2019). Habitat Loss Does Not Always Entail Negative Genetic Consequences. Front. Genet..

[182] Willi Y., Hoffmann A.A. (2009). Demographic Factors and Genetic Variation Influence Population Persistence under Environmental Change. J. Evol. Biol..

[183] Barghi N., Hermisson J., Schlötterer C. (2020). Polygenic Adaptation: A Unifying Framework to Understand Positive Selection. Nat. Rev. Genet..

[184] Tigano A., Friesen V.L. (2016). Genomics of Local Adaptation with Gene Flow. Molec. Ecol..

[185] Robert A. (2009). Captive Breeding Genetics and Reintroduction Success. Biol. Conserv..

[186] Calatayud N.E., Jacobs L., Della Togna G., Langhorne C.J., Mullen A.C., Upton R. (2023). Hormonal Induction, Quality Assessments and the Influence of Seasonality on Male Reproductive Viability in a Long-term Managed Ex Situ Breeding Colony of Southern Rocky Mountain Boreal Toads, *Anaxyrus boreas boreas*. bioRxrv.

[187] Edmands S. (2007). Between a Rock and a Hard Place: Evaluating the Relative Risks of Inbreeding and Outbreeding for Conservation and Management. Mol. Ecol..

[188] Earnhardt J.M. (2006). Reintroduction Programmes: Genetic Trade-offs for Populations. Anim. Conserv..

[189] Byrne P.G., Silla A.J., Silla A.J., Kouba A.J., Heatwole H. (2022). Genetic Management of Threatened Amphibians: Using Artificial Fertilisation Technologies to Facilitate Genetic Rescue and Assisted Gene Flow. Reproductive Technologies and Biobanking for the Conservation of Amphibians.

[190] Byrne P.G., Keogh J.S., O’Brien D.M., Gaitan-Espitia J.D., Silla A.J. (2021). Evidence that Genetic Compatibility Underpins Female Mate Choice in a Monandrous Amphibian. Evolution.

[191] Spielman D., Brook B.W., Frankham R. (2004). Most Species are not Driven to Extinction before Genetic Factors Impact Them. Proc. Nat. Acad. Sci. USA.

[192] Teixeira J.C., Huber C.D. (2021). The Inflated Significance of Neutral Genetic Diversity in Conservation Genetics. Proc. Nat. Acad. Sci. USA.

[193] Walter H.S. (2004). The Mismeasure of Islands: Implications for Biogeographic Theory and the Conservation of Nature. J. Biogeogr..

[194] Revive and Restore The Black-Footed Ferret Project. https://reviverestore.org/projects/black-footed-ferret/.

[195] Scheele B.C., Guarino F., Osborne W., Hunter D.A., Skerratt L.F., Driscoll D.A. (2014). Decline and Re-expansion of an Amphibian with High Prevalence of Chytrid Fungus. Biol. Conserv..

[196] O’Hanlon S.J., Rieux A., Farrer R.A., Rosa G.M., Waldman B., Bataille A., Kosch T.A., Murray K.A., Brankovics B., Fumagalli M. (2018). Recent Asian Origin of Chytrid Fungi Causing Global Amphibian Declines. Science.

[197] Dreitz V.J. (2006). Issues in Species Recovery: An Example Based on the Wyoming Toad: Forum. BioScience.

[198] West M., Todd C.R., Gillespie G.R., McCarthy M. (2020). Recruitment is Key to Understanding Amphibian’s Different Population-level Responses to Chytrid Fungus Infection. Biol. Conserv..

[199] Atkinson M.S., Anna E., Savage A.E. (2023). Invasive amphibians alter host-pathogen interactions with primarily negative outcomes for native species. Biol. Conserv..

[200] Viggers K.L., Lindenmayer D.B., Spratt D.M. (1993). The Importance of Disease in Reintroduction Programmes. Wildl. Res..

[201] Ballou J.D. (1993). Assessing the Risks of Infectious Diseases in Captive Breeding and Reintroduction Programs. J. Zoo Wildl. Med..

[202] Chaudhary V., Oli M.K. (2019). A Critical Appraisal of Population Viability Analysis. Conserv. Biol..

[203] Willi Y., Kristensen T.N., Sgrò C.M., Weeks A.R., Ørsted M., Hoffmann A.A. (2022). Conservation genetics as a management tool: The Five Best-supported Paradigms to Assist the Management of Threatened Species. Proc Natl Acad Sci USA.

[204] Frankham R. (1997). Do Island Populations Have Less Genetic Variation than Mainland Populations?. Heredity.

[205] Fauvel C., Suquet M., Cosson J. (2010). Evaluation and Determinism of Fish Sperm Quality. J. Appl. Ichthyol..

[206] EDGE. Evolutionary Distinct and Globally Endangered Zoological Society of London. https://www.edgeofexistence.org/species/.

[207] Khosla K., Kangas J., Liu Y., Zhan L., Daly J., Hagedorn M., Bischof J. (2020). Cryopreservation and Laser Nanowarming of Zebrafish Embryos Followed by Hatching and Spawning. Adv. Biosyst..

[208] de Siqueira-Silva D.H., Saito T., dos Santos-Silva A.P., da Silva Costa R., Psenicka M., Yasui G.S. (2018). Biotechnology Applied to Fish Reproduction: Tools for Conservation. Fish Physiol. Biochem..

[209] Browne R.K., Figiel C., Tiersch T., Mazik P. (2011). Chapter 2. Crypreservation in Amphibians. Cryopreservation of Aquatic Species.

[210] Behera B.J., Behera B.K. (2024). Surrogacy Technology in Fisheries and Aquaculture. Current Trends in Fisheries Biotechnology.

[211] Browne R.K., Zippel K., Odum A.R., Herman T. (2007). Physical Facilities and Associated Services. Use of Amphibians in Research, Laboratory, or Classroom Settings. Inst. Lab. Anim. Res. (ILAR).

[212] AArk Reproduction Technologies Videos. https://www.amphibianark.org/art-videos/.

[213] Hamer A.J., Mahony M.J. (2007). Life History of an Endangered Amphibian Challenges the Declining Species Paradigm. Aust. J. Zool..

[214] Richter S.C., Seigel R.A. (2002). Annual Variation in the Population Ecology of the Endangered Gopher Frog, *Rana sevosa* Goin and Netting. Copeia.

[215] McGinnity D., Reinsch S.R., Schwartz H., Trudeau V., Browne R.K. (2021). Semen and Egg Collection, Sperm Cryopreservation, and In Vitro Fertilisation with Threatened North American Giant Salamanders (*Cryptobranchus alleganiensis*). Reprod. Fertil. Dev..

[216] Lötters S., Plewnia A., Catenazzi A., Neam K., Acosta-Galvis A.R., Vela Y.A., Allen J.P., Segundo J.O.A., de Lourdes Almendáriz Cabezas A., Barboza G.A. (2023). Ongoing Harlequin Toad Declines Suggest the Amphibian Extinction Crisis is Still an Emergency. Commun. Earth Environ..

[217] Valencia L.M., Fonte L.F. (2007). The Fate of Harlequin Toads—Help through a Synchronous Approach and the IUCN ‘Amphibian Conservation Action Plan’?. Oryx.

[218] Valencia L.M., Fonte L.F. (2021). Atelopus Survival Initiative IUCN SSC ASG Atelopus Task Force. https://www.atelopus.org/_files/ugd/9db650_60f3e6095cbf4b1dabb7376a4fb88366.pdf.

[219] Buckner J.C., Sanders R.C., Faircloth B.C., Chakrabarty P. (2021). The Critical Importance of Vouchers in Genomics. eLife.

[220] Andreone D., Raselimanana A.P., Crottini A. (2023). Vouchering, Integrative Taxonomy and Natural History Collections: A Case Study with the Amphibians of Madagascar. Boll. Mus. Reg. Sci. Nat. Tor..

[221] Rocha L.A., Aleixo A., Allen G., Almeda F., Baldwin C.C., Barclay M.V.L., Bates M., Bauer A.M., Benzoni F., Berns C.M. (2014). Specimen Collection: An Essential Tool. Science.

[222] Li Y., Hopkins A.J.M., Davis R.A. (2023). Going, Going, Gone. The Diminishing Capacity of Museum Specimen Collections to Address Global Change Research: A Case Study on Urban Reptiles. Animals.

[223] Powell D.M., Meyer T.G., Duncan M. (2023). By Bits and Pieces: The Contributions of Zoos and Aquariums to Science and Society via Biomaterials. J. Zool. Bot. Gard..

[224] Goncharov B.F., Shubravy O.I., Serbinova I.A., Uteshev V.K. (1989). The USSR Programme for Breeding Amphibians, Including Rare and Endangered Species. Int. Zoo Yearb..

[225] Rakesh R.K. (1976). Seasonal Cycle in Anuran (Amphibia) Testis: The Endocrine and Environmental Controls. Ital. J. Zool..

[226] Rastogi R.K., Tammaro L., Di Meglio M., Lela L., Di Matteo l., Giovanni Chieffi G. (1981). Circannual Testicular Rhythm in the Green Frog, *Rana esculenta*. Ital. J. Zool..

[227] Ulloa J.S., Aubin T., Llusia D., Courtois É.A., Fouquet A., Gaucher P., Pavoine S., Sueur J. (2019). Explosive Breeding in Tropical Anurans: Environmental Triggers, Community Composition and Acoustic Structure. BMC Ecol..

[228] Kaurova S.A., Uteshev V.K., Gapeyev A.B., Shishova N.V., Gakhova E.N., Browne R.K., Kramarova L.I. (2021). Cryopreservation of Spermatozoa Obtained Postmortem from the European Common Frog *Rana temporaria*. Reprod. Fertil. Dev..

[229] Figiel C.R. (2023). Effects of Water Temperature on Gonads Growth in *Ambystoma mexicanum* Axolotl Salamanders. Animals.

[230] O’Brien D.M., Silla A.J., Forsythe P.S., Byrne P.G. (2021). Sex Differences in Response to Environmental and Social Breeding Cues in an Amphibian. Behaviour.

[231] Edwards D.L., Mahony M.J., Clulow J. (2004). Effect of Sperm Concentration, Medium Osmolality and Oocyte Storage on Artificial Fertilisation Success in a Myobatrachid Frog (*Limnodynastes tasmaniensis*). Reprod. Fertil. Dev..

[232] Upton R., Clulow S., Colyvas K., Mahony M., Clulow J. (2023). Paradigm Shift in Frog Sperm Cryopreservation: Reduced Role for Non-penetrating Cryoprotectants. Reproduction.

[233] Upton R., Clulow S., Mahony M.J., and Clulow J. (2018). Generation of a Sexually Mature Individual of the Eastern Dwarf Tree Frog *Litoria fallax*, from Cryopreserved Testicular Macerates: Proof of Capacity of Cryopreserved Sperm Derived Offspring to Complete Development. Conserv. Physiol..

[234] Silla A.J., Keogh L.M., Byrne P.G. (2017). Sperm Motility Activation in the Critically Endangered Booroolong Frog: The Effect of Medium Osmolality and Phosphodiesterase Inhibitors. Reprod. Fertil. Dev..

[235] Germano J.M., Cree A., Molinia F.C., Arregui L., Bishop P.J. (2021). Hormone Treatment does not Reliably Induce Spermiation or Mating in Hamilton’s frog from the Archaic Leiopelmatid Lineage. Reprod. Fertil. Dev..

[236] Hormone Therapy. https://en.wikipedia.org/wiki/Hormone_therapy.

[237] Browne R.K., Wang Z., Okada S., McGinnity D., Luo Q., Taguchi Y., Kilpatrick D., Hardman R., Janzen P., Zhang Z. (2020). The Sustainable Management of Giant Salamanders (Cryptobranchoidea).

[238] Miyamoto K., Simpson D., Gurdon J.B. (2015). Manipulation and In vitro Maturation of *Xenopus laevis* Eggs, Followed by Intracytoplasmic Sperm Injection, to Study Embryonic Development. J. Vis. Exp..

[239] Herbert D. (2004). Studies of Assisted Reproduction in the Spotted Grass Frog *Limnodynastes tasmaniensis*: Ovulation, Early Development and Microinjection (ICSI). Master’s Thesis.

[240] Ishibashi S., Kroll K.L., Amaya E. (2007). Generation of Transgenic *Xenopus laevis*: III. Sperm Nuclear Transplantation. Cold Spring Harb. Protoc..

[241] Parmar M.S., Pant C., Karuppanasamy K., Mili B., Upadhyay D., Kant V. (2013). Intracytoplasmic Sperm Injection (ICSI) and its Applications in Veterinary Sciences: An Overview. Sci. Int..

[242] Silla A.J., Byrne P.G. (2021). Hormone-induced Ovulation and Artificial Fertilisation in Four Terrestrial-breeding Anurans. Reprod. Fertil. Dev..

[243] Browne R.K., Seratt J., Vance C., Kouba A. (2006). Hormonal Priming, Induction of Ovulation and *In-vitro* Fertilization of the Endangered Wyoming Toad (*Bufo baxteri*). Reprod. Biol. Endocrinol..

[244] Marcec-Greaves R.M., Kouba C.K., Willard S.T., Andy J., Kouba A.J. (2023). Ovarian Ultrasound Analysis for Developing Temporal and Spatially Explicit Hormone Regimens for Induced Ovulation and Egg Deposition in the Tiger Salamander (*Ambystoma tigrinum*). Theriogenology Wild.

[245] Shishova N.V., Uteshev V.K., Sirota N.P., Kuznetsova E.A., Kaurova S.A., Browne R.K., Gakhova E.N. (2013). The Quality and Fertility of Sperm Collected from European Common Frog (*Rana temporaria*) Carcasses Refrigerated for up to 7 Days. Zoo Biol..

[246] Silla A.J., Roberts J.D., Byrne P.G. (2020). The Effect of Injection and Topical Application of hCG and GnRH Agonist to Induce Sperm-release in the Roseate Frog, *Geocrinia rosea*. Conserv. Physiol..

[247] Marcec R.M. (2016). Development of Assisted Reproductive Technologies for Endangered North American Salamanders by Ruth Marie Marcec. Ph.D. Thesis.

[248] Coxe N., Liu Y., Arregui L., Upton R., Bodenstein S., Voss S.R., Gutierrez-Wing M.T., Tiersch T.R. (2024). Establishment of a Practical Sperm Cryopreservation Pathway for the Axolotl (*Ambystoma mexicanum*): A Community-Level Approach to Germplasm Repository Development. Animals.

[249] Guy E.L., Gillis A.B., Kouba A.J., Barber D., Poole V., Marcec-Greaves R.M., Kouba C.K. (2020). Sperm Collection and Cryopreservation for Threatened Newt Species. Cryobiology.

[250] Chen D.M., Kouba C.K., Songsasen N., Roth T.L., Allen P.J., Kouba A.J. (2023). Comparing Novel Sperm Extenders for the Internally-fertilizing Tiger Salamander (*Ambystoma tigrinum*). Front. Amphib. Reptile Sci..

[251] Julien A.R., Kouba A.J., Kabelik D., Feugangm J.M., Willard S.T., Kouba C.K. (2019). Nasal Administration of Gonadotropin Releasing Hormone (GnRH) Elicits Sperm Production in Fowler’s Toads (*Anaxyrus fowleri*). BMC Zool..

[252] Campbell L.G., Anderson K.A., Marcec-Greaves R.M. (2021). Topical Application of Hormone Gonadotropin-releasing Hormone (GnRH-A) Stimulates Reproduction in the Endangered Texas Blind Salamander (*Eurycea rathbuni*). Conserv. Sci. Pract..

[253] Toledo R.C., Jared C. (1993). Cutaneous Adaptations to Water Balance in Amphibians. Comp. Biochem. Physiol. B Biochem. Physiol..

[254] Chen D.M., Chen L.D., Kouba C.K., Songsasen N., Roth T.L., Allen P.J., Kouba A.J. (2024). Oral Administration of GnRH via a Cricket Vehicle Stimulates Spermiation in Tiger Salamanders (*Ambystoma tigrinum*). PLoS ONE.

[255] Clulow J., Pomering M., Herbert D., Upton R., Calatayud N., Clulow S., Mahony M.J., Trudeau V.L. (2018). Differential success in obtaining gametes between male and female Australian temperate frogs by hormonal induction: A review. Gen. Comp. Endocrinol..

[256] Trudeau V.L., Raven B.H., Pahuja H.K., Narayan E.J., Silla A.J., Kouba A.J., Heatwole H. (2022). Chapter 4. Hormonal Control of Amphibian Reproduction. Reproductive Technologies and Biobanking for the Conservation of Amphibians.

[257] Browne R.K., Li H., Seratt J., Kouba A. (2006). Progesterone Improves the Number and Quality of Hormone Induced Fowler toad (*Bufo fowleri*) Oocytes. Reprod. Biol. Endocrinol..

[258] Mansour N., Lahnsteiner F., Patzner R.A. (2011). Collection of Gametes from Live Axolotl, *Ambystoma mexicanum*, and Standardization of In Vitro Fertilization. Theriogenology.

[259] Uteshev V.K., Kaurova S.A., Shishova N.V., Stolyarov S.D., Browne R.K., Gakhova E.N. (2015). In Vitro Fertilization with Hormonally Induced Sperm and Eggs from Sharp-ribbed Newt *Pleurodeles waltl*. Russ. J. Herpetol..

[260] Graham K.M., Langhorne C.J., Vance C.K., Willard S.T., Kouba A.J. (2018). Ultrasound Imaging Improves Hormone Therapy Strategies for Induction of Ovulation and In Vitro Fertilization in the Endangered Dusky Gopher Frog (*Lithobates sevosa*). Conserv. Physiol..

[261] Bogolyubova I.O., Bogolyubov D.S. (2023). Effect of Hormonal Stimulation on the Oocyte Chromosomal Apparatus in the Common Frog. J. Evol. Biochem. Phys..

[262] Silla A.J., Langhorne C.J., Silla A.J., Kouba A.J., Heatwole H. (2022). Protocols for Hormonally Induced Spermiation, and the Cold Storage, Activation, and Assessment of Sperm. Reproductive Technologies and Biobanking for the Conservation of Amphibians.

[263] Burger I.J., Chen L., Lampert S.S., Kouba C.K., Barber D., Smith D., Cobos C., Kouba A.J. (2023). Applying Sperm Collection and Cryopreservation Protocols Developed in a Model Amphibian to Three Threatened Anuran Species Targeted for Biobanking Management. Biol. Conserv..

[264] Arregui L., Martinez-Pastor F., Arroyo F., Gosálvez J. (2022). Determining the Effects of Sperm Activation in Anuran Cloaca on Motility and DNA Integrity in *Epidalea calamita* (Bufonidae). Reprod. Fertil. Dev..

[265] Valencia L.C., García A., Ramírez-Pinilla M.P., Fuentes J.L. (2011). Estimates of DNA Damage by the Comet Assay in the Direct-developing Frog *Eleutherodactylus johnstonei* (Anura, Eleutherodactylidae). Genet. Mol. Biol..

[266] Shishova N.V., Uteshev V.K., Kaurova S.A., Browne R.K., Gakhova E.N. (2011). Cryopreservation of Hormonally Induced Sperm for the Conservation of Threatened Amphibians with *Rana temporaria* as a Model Research Species. Theriogenology.

[267] Tanga B.M., Qamar A.Y., Raza S., Bang S., Fang X., Yoon K., Cho J. (2021). Semen Evaluation: Methodological Advancements in Sperm Quality-specific Fertility Assessment—A Review. Anim. Biosci..

[268] Krapf D., Visconti P.E., Arranz S.E., Cabada M.O. (2007). Egg Water from the Amphibian *Bufo arenarum* Induces Capacitation-Like Changes in Hhomologous Spermatozoa. Dev. Biol..

[269] Jorge-Neto P.N., de Moraes Francisco F., Carneiro M.D.D., Santos S.R.B., Requena L.A., Ramos S.D., de Goés M.F., Valle R.F., Padilha F.L.A., Colbachini H. (2024). Specific Setup and Methodology for Computer-assisted Sperm Analysis (CASA) in Evaluating Elasmobranch Sperm. Theriogenology Wild.

[270] Hobbs R.J., Upton R., Calatayud N.E., Silla A.J., Daly J., McFadden M.S., O’Brien J.K. (2023). Cryopreservation Cooling Rate Impacts Post-thaw Sperm Motility and Survival in *Litoria booroolongensis*. Animals.

[271] Browne R.K., Venu G., Kaurova S.A. (2023). The Case for Considering the Term ‘Mitochondrial Vesicle’ as a Misnomer in Publications about Assisted Reproductive Technologies (ART) for Amphibians. Reprod. Fertil. Dev..

[272] Lee M.S.Y., Jamieson B.G.M. (1993). The Ultrastructure of the Spermatozoa of Bufonid and Hylid Frogs (Anure, Amphibia): Implications for Phylogeny and Fertilisation Biology. Zool. Scr..

[273] Chen D.M., Moore M.G., Willis E.L., Kouba A.J., Kouba C.K. (2022). The Impact of Time and Environmental Factors on the Mitochondrial Vesicle and Subsequent Motility of Amphibian Sperm. Comp. Biochem. Physiol. A Mol. Integr. Physiol..

[274] Shishova N.V., Kaurova S.A., Uteshev V.K., Gakhova E.N. (2023). Comparison of Cryoresistance of Testicular and Urinal Spermatozoa of the Toad *Bufo bufo* (Amphibia, Anura, Bufonidae) during Slow Freezing. Inland Water Biol..

[275] Figiel C.R. (2022). Cold Storage of Sperm from the Axolotl, *Ambystoma mexicanum*. Herpetol. Conserv. Biol..

[276] Silla A.J., Keogh L.M., Byrne P.G. (2015). Antibiotics and Oxygen Availability Affect the Short-term Storage of Spermatozoa from the Critically Endangered Booroolong Frog, *Litoria booroolongensis*. Reprod. Fertil. Dev..

[277] Germano J.M., Arregui L., Kouba A.J. (2013). Effects of Aeration and Antibiotics on Short-term Storage of Fowler’s toad (*Bufo fowleri*) Sperm. Aquaculture.

[278] Kaurova S.A., Browne R.K., Uteshev V.K. (2022). Antibiotics for the Refrigerated Storage at 4 °C of Hormonally Induced European Common Frog (*Rana temporaria*) Spermatozoa. Theriogenology Wild.

[279] Kaurova S.A., Shishova N.V., Uteshev V.K. (2023). The Effect of Gentamicin on the Motility of Hormonally Induced Spermatozoa of Toad *Bufo bufo* during Storage at 4 °C. Bull. Exp. Biol. Med..

[280] Kaurova S.A., Uteshev V.K., Shishova N.V. (2024). Effect of Antibiotics Metranidazole, Streptomycin, and Gentamicin on the Maintenance of Sperm Motility of the European Common Frog (*Rana temporaria*) During Refrigerated Storage. Russ. J. Herpetol..

[281] Arregui L., Bóveda P., Gosálvez J., Kouba A.J. (2020). Effect of Seasonality on Hormonally Induced Sperm in *Epidalea calamita* (Amphibia, Anura, Bufonidae) and its Refrigerated and Cryopreserved Storage. Aquaculture.

[282] Uteshev V.K., Gakhova E.N., Kramarova L.I., Shishova N.V., Kaurova S.A., Browne R.K. (2018). Refrigerated Storage of European Common Frog *Rana temporaria* Oocytes. Cryobiology.

[283] Poo S., Hinkson K.M. (2019). Applying Cryopreservation to Anuran Conservation Biology. Conserv. Sci. Pract..

[284] Kaneko T., Ito H., Sakamoto H., Onuma M., Inoue-Murayama M. (2014). Sperm Preservation by Freeze-drying for the Conservation of Wild Animals. PLoS ONE.

[285] Liu M., Yuan S., Zhao Z., Liu M., Lin A., Gong Q. (2021). Non-programmable Cryopreservation of Sperm from Industrial-farmed Murray Cod (*Maccullochella peelii*). Aquaculture.

[286] Silla A.J., Kouba A.J., Silla A.J., Kouba A.J., Heatwole H. (2022). Chapter 1. Integrating Reproductive Technologies into the Conservation Toolbox for the Recovery of Amphibian Species. Reproductive Technologies and Biobanking for the Conservation of Amphibians.

[287] Clulow J., Upton R., Clulow S., Silla A.J., Kouba A.J., Heatwole H. (2022). Cryopreservation of Amphibian Genomes: Targeting the Holy Grail, Cryopreservation of Maternal-haploid and Embryonic-Diploid Genomes. Reproductive Technologies and Biobanking for the Conservation of Amphibians.

[288] Wolf D.P., Hendrick J.L. (1971). A Molecular Approach to Fertilisation. II. Viability and Artificial Fertilisation of *Xenopus laevis* Gametes. Dev. Biol..

[289] Hollinger T.G., Corton G.L. (1980). Artificial Fertilization of Gametes from the South African Clawed Frog, *Xenopus laevis*. Gamete Res..

[290] Gagarinskiy E., Uteshev V., Fesenko E. (2023). Prolonged hypothermic storage of oocytes of the European Common Frog *Rana temporaria* in a Gas Mixture of Oxygen and Carbon Monoxide. PLoS ONE.

[291] Eberhard W. (1966). Female Control: Sexual Selection by Cryptic Female Choice.

[292] Ukita M., Itoh T., Watanabe T., Watanabe A., Onitake K. (1999). Substances for the Initiation of Sperm Motility in Egg-jelly of the Japanese Newt, *Cynops pyrrhogaster*. Zool. Sci..

[293] Olson J.H., Chandler D.E. (1999). *Xenopus laevis* Egg Jelly contains Small Proteins that are essential to fertilization. Dev. Biol..

[294] Omata S. (1993). Relative Roles of Jelly Layers in Successful Fertilization of *Bufo japonicus*. J. Exp. Zool..

[295] Simmons L.W., Roberts J.D., Dziminski M.A. (2009). Egg Jelly Influences Sperm Motility in the Externally Fertilizing Frog, *Crinia georgiana*. Evol. Biol..

[296] Comizzoli P. (2015). Biotechnologies for Wildlife Fertility Preservation. Anim. Front..

[297] Briggs R., King T.J. (1952). Transplantation of Living Nuclei from Blastula Cells into Enucleated Frogs’ Eggs. Proc. Natl. Acad. Sci. USA.

[298] Sambuichi H. (1957). The Roles of the Nucleus and the Cytoplasm in Development. I. An Interspecific Hybrid Frog, Developed from a Combination of *Rana nigromaculata nigromaculata* Cytoplasm and a Diploid Nucleus of *Rana nigromacula tabrevipoda*. J. Sci. Hiroshima Univ..

[299] Moore J.A. (1958). Transplanation of Nuclei between *Rana pipiens* and *Rana sylvatica*. Exp. Cell Res..

[300] Sambuichi H. (1961). The Roles of the Nucleus and the Cytoplasm in Development. III. Diploid Nucleocytoplasmic Hybrids, Derived from *Rana nigromaculata brevipoda* Cytoplasm and *Rana nigromaculata nigromaculata* Nuclei. J. Sci. Hiroshima Univ..

[301] Hennan S. (1963). Nucleocytoplasmic Hybrids between *Rana pipiens* and *Rana palustris*. I. Analysis of the Developmental Properties of the Nuclei by Means of Nuclear Transplantation. Dev. Biol..

[302] Toshijiro K., Nishioka M. (1972). Viability and Abnormalities of the Offspring of Nucleo-cytoplasmic Hybrids between *Rana japonica* and *Rana ornativentris*. Sci. Rep. Lab. Amphib. Biol. Hiroshima Univ..

[303] Nishioka M. (1972). Abnormalities of the Offspring of Nucleo-cytoplasmic Hybrids between *Rana nigromaculata* and *Rana brevipoda*. Sci. Rel. Lab. Amphib. Biol. Hiroshima Univ..

[304] Nishioka M. (1972). Nucleo-cytoplasmic Hybrids between *Rana japonica* and *Rana temporaria temporaria*. Sci. Rep. Lab. Amphib. Biol. Hiroshima Univ..

[305] Nishioka M. (1972). Nucleo-cytoplasmic Hybrids between *Rana brevipoda* and *Rana plancyichosenica*. Rep. Lab. Amphib. Biol. Hiroshima Univ..

[306] Gallien L., Aimar C. (1972). On a New Mode of Gemelarity, Produced by Nuclear Grafting in Amphibians (Urodeles) of the Genus *Pleurodeles*. Acad. Sci..

[307] Gallien C.L., Aimar C., Guillet F. (1973). Nucleocytoplasmic Interactions During Ontogenesis in Individuals Obtained by Intra- and Interspecific Nuclear Transplantation in the Genus *Pleurodeles* (Urodele Amphibian). Dev. Biol..

[308] Kouba A., Vance C., Willis E. (2009). Artificial Fertilization for Amphibian Conservation: Current Knowledge and Future Considerations. Theriogenology.

[309] Taniguchi-Sugiura Y., Tanaka E.M. (2023). Artificial Insemination in Axolotl. Methods Mol. Biol..

[310] Rugh R. (1962). Experimental Embryology: Techniques and Procedures.

[311] Hoitsy G.A., Woynarovich T., Moth-Poulsen T. (2012). Guide to the Small-Scale Artificial Propagation of Trout.

[312] Rugh R. (1934). Induced Ovulation and Artificial Fertilisation in the Frog. Biol. Bull..

[313] Vasilescu S.A., Ding L., Parast F.Y., Nosrati R., Warkiani M.E. (2023). Sperm Quality Metrics were Improved by a Biomimetic Microfluidic Selection Platform Compared to Swim-up Methods. Microsyst. Nanoeng..

[314] Cabada M.O. (1975). Sperm Concentration and Fertilisation Rate in *Bufo arenarum* (Amphibia: Anura). J. Exp. Biol..

[315] Silla A.J. (2013). Artificial Fertilisation in a Terrestrial Toadlet (*Pseudophryne guentheri*): Effect of Medium Osmolarity, Sperm Concentration and Gamete Storage. Reprod. Fertil. Dev..

[316] Byrne P.G., Anastas Z.M., Silla A.J. (2022). A Test for Plasticity in Sperm Motility Activation in Response to Osmotic Environment in an Anuran Amphibian. Ecol Evol..

[317] Dcunha R., Hussein R.S., Ananda H., Kumari S., Adiga S.K., Kannan N., Zhao Y., Kalthur G. (2022). Current Insights and Latest Updates in Sperm Motility and Associated Applications in Assisted Reproduction. Reprod. Sci..

[318] Browne R.K., Kaurova S.A., Uteshev V.K., Shishova N.V., Kramarova L., McGinnity D., Figiel C.R., Mansour N., Agnew D., Wu M. (2015). Sperm Motility of Externally Fertilizing Fish and Amphibians. Theriogenology.

[319] Burger I.J., Lampert S.S., Kouba C.K., Morin D.J., Kouba A.J. (2022). Development of an Amphibian Sperm Biobanking Protocol for Genetic Management and Population Sustainability. Conserv. Physiol..

[320] Strand J., Callesen H., Pertoldi C., Purupm S. (2022). Amphibian cell lines: Usable tissue types and differences between individuals within a species. Amphib. Reptile Conserv..

[321] Nikitina L.A. (1997). Nuclear Transplantation in Amphibians. Physiol. Gen. Biol. Rev..

[322] Kaurova S.A., Nikitina L.A., Uteshev V.K., Gakhova E.N., Gakhova E., Karnaukhov V.N. (1998). Cryopreservation of Totipotent Embryo Cells and Their use in Reconstruction of Enucleated Eggs. Proceedings of the 15th Working Meeting.

[323] De-Extinction. https://colossal.com/de-extinction/.

[324] Gurdon J.B. (1962). Adult Frogs Derived from the Nuclei of Single Somatic Cells. Dev. Biol..

[325] (2012). The Nobel Prize in Physiology or Medicine. https://www.nobelprize.org/prizes/medicine/2012/summary/.

[326] Gurdon J.B., Byrne J.A. (2003). The First Half-century of Nuclear Transplantation. Proc. Natl. Acad. Sci. USA..

[327] Gurdon J.B. (2013). The Egg and the Nucleus: A Battle for Supremacy. Development.

[328] Zeller U., Thomas Göttert T. (2019). The Relations Between Evolution and Domestication Reconsidered - Implications for systematics, Ecology, and Nature Conservation. Glob. Ecol. Conserv..

[329] Kosch T.A., Silva C.N.S., Brannelly L.A., Roberts A.A., Lau Q., Marantelli G., Berger L.F.L., Skerratt L.F. (2019). Genetic Potential for Disease Resistance in Critically Endangered Amphibians Decimated by Chytridiomycosis. Anim. Conserv..

[330] Temple J. (2024). How a breakthrough gene-editing tool will help the world cope with climate change. MIT Technol. Rev..

[331] World Animal Protection. https://www.worldanimalprotection.org/.

[332] Beaulieu M. (2024). Capturing Wild Animal Welfare: A Physiological Perspective. Biol. Rev..

[333] Singer P. 10th Anniversary Edition of The Life You Can Save. https://www.thelifeyoucansave.org/the-book/.

[334] Irwin L.N. (2020). Renewed Perspectives on the Deep Roots and Broad Distribution of Animal Consciousness. Front. Syst. Neurosci..

[335] Lambert H., Elwin A., D’Cruze N. (2022). Frog in the Well: A Review of the Scientific Literature for Evidence of Amphibian Sentience. Appl. Anim. Behav. Sci..

[336] Winlow W., Mather J., Cosmo A.D. (2024). Postscript to Invertebrate Welfare: “We Have Met the Enemy and He Is Us”. Animals.

[337] Andrews K., Birch J., Sebo J., Sims T. (2024). Background to the New York Declaration on Animal Consciousness. https://sites.google.com/nyu.edu/nydeclaration/declaration.

[338] Offor I. (2020). Second Wave Animal Ethics and (Global) Animal Law: A View from the Margins. J. Hum. Rights Environ..

[339] Ikegbu E.A., Diana-Abasi F.I. (2017). Utilitarianism as a Veritable Vehicle for the Promotion of a Just Society. LWATI J. Contemp. Res..

[340] Russell W.M.S., Burch R.L. (1959). The Principles of Humane Experimental Technique.

[341] Tannenbaum J., Bennett B.T. (2015). Russell and Burch’s 3Rs Then and Now: The Need for Clarity in Definition and Purpose. J. Am. Assoc. Lab. Anim. Sci..

[342] NC3R National Center for the Replacement, Reduction, and Refinement of Animals in Research. https://www.nc3rs.org.uk/who-we-are/3rs.

[343] Silla A.J., Calatayud N.E., Trudeau V.L. (2021). Amphibian Reproductive Technologies: Approaches and Welfare Considerations. Conserv. Physiol..

[344] Woolly Mammoth De-Extinction Project & Process|Colossal. https://colossal.com/mammoth/.

[345] van Urk-Costa E. (2023). Introduction—Why (Reformed) Theology Needs Reflection on Biodiversity Loss and Extinction. J. Reform. Theol..

[346] Link H.J. (2013). Playing God and the Intrinsic Value of Life: Moral Problems for Synthetic Biology?. Sci. Eng. Ethics.

[347] Dabrock P. (2009). Playing God? Synthetic Biology as a Theological and Ethical Challenge. Syst. Synth. Biol..

[348] Peters T. (1995). Playing God” and Germline Intervention. Appl. Anim. Behav. Sci..

[349] Negi C.S. (2005). Religion and Biodiversity Conservation: Not a Mere Analogy. Int. J. Biodiv. Sci. Manag..

[350] Jamieson B.G.M., Exbrayat J., Jamieson B.G.M. (2006). Reproductive Biology and Phylogeny of Gymnophiona. Volume 5 of a Series Reproductive Biology and Phylogeny Caecilians.

[351] White T.B., Petrovan S.O., Christie A.P., Martin P.A., Sutherland W.J. (2022). What is the Price of Conservation? A Review of the Status Quo and Recommendations for Improving Cost Reporting. BioScience.

[352] Rowling M. How Much Will It Cost to Save Nature—And Who Will Pay? World Economic Forum and the Thomas Reuters Foundation Trust.org. https://www.weforum.org/agenda/2021/05/biodiversity-deforestation-global-investment-inititive/.

[353] Gordon E.R., Butt N., Rosner-Katz H., Binley A.D., Bennett J.R. (2019). Relative Costs of Conserving Threatened Species Across Taxonomic Groups. Conserv. Biol..

[354] Balmford A., Gaston K.J., Rodrigues A.S.L., James A. (2013). Integrating Costs of Conservation into International Priority Setting. Conserv. Biol..

[355] Conde-Pueyo N., Vidiella B., Sardanyés J., Berdugo M., Maestre F.T., De Lorenzo V., Solé R. (2020). Synthetic Biology for Terraformation Lessons from Mars, Earth, and the Microbiome. Life.

[356] Gualandris-Parisot L., Husson D., Foulquier F., Kan P., Davet J., Aimar C., Dournon C., Duprat A.M. (2001). *Pleurodeles waltl*, Amphibian, Urodele, is a Suitable Biological Model for Embryological and Physiological Space Experiments on a Vertebrate. Adv. Space Res..

[357] Jacobson S.K., McDuff M.D., Monroe M.C. (2007). Conservation Education and Outreach Techniques. Techniques in Ecology & Conservation.

[358] Meredith H.M.R., St. John F.A.V., Collen B., Black S.A., Griffiths R.A. (2018). Practitioner and Scientist Perceptions of Successful Amphibian Conservation. Conserv. Biol..

[359] Bradfield K.S., Tapley B., Johnson B. (2023). Amphibians and Conservation Breeding Pro-grammes: How do we Determine Who Should be on the Ark?. Biodiver. Conserv..

[360] The Nature Education Knowledge Project Saving Endangered Species: A Case Study Using Global Amphibian Declines. https://www.nature.com/scitable/knowledge/library/saving-endangered-species-a-case-study-using-19445898/.

[361] Rothschild J. (2020). Ethical Considerations of Gene Editing and Genetic Selection. J. Gen. Fam. Med..

[362] Frieze C. (2013). Cloning Wild Life—Zoos, Captivity, and the Future of Endangered Animals.

[363] Voigt C.A. (2020). Synthetic Biology 2020–2030: Six Commercially-available Products that are Changing Our World. Nat. Commun..

[364] Meng F., Ellis T. (2020). The Second Decade of Synthetic Biology: 2010–2020. Nat. Commun..

[365] Loy I., Worley W. (2023). Your Jargon-Busting Climate Glossary. https://www.thenewhumanitarian.org/news-feature/2023/12/01/oh-ffs-guide-climate-change-acronyms.

[366] Charo A. Charo: Bioengineering Can Help Heal the World. https://colossal.com/charo-bioengineering-can-help-heal-the-world/.

[367] Lann B. Petition to the United Federation of Planets: Revert the Ban on Gene Editing for Ecological Applications. https://www.reddit.com/r/startrek/comments/1df045s/petition_to_the_united_federation_of_planets/?rdt=37953.

[368] Schwägerl C. (2015). The Anthropocene—The Human Era and How It Shapes the Planet.

[369] Malhi Y. (2017). The Concept of the Anthropocene. Ann. Rev. Environ. Resour..

[370] Tapley B., Bradfield B., Michaels C., Bungard M. (2015). Amphibians and Conservation Breeding Programmes: Do all Threatened Amphibians Belong on the Ark?. Biodivers. Conserv..

